# Unraveling genetic variation among white spruce families generated through different breeding strategies: Heritability, growth, physiology, hormones and gene expression

**DOI:** 10.3389/fpls.2023.1052425

**Published:** 2023-04-03

**Authors:** Esteban Galeano, Barb R. Thomas

**Affiliations:** ^1^ Department of Forestry, Mississippi State University, Starkville, MS, United States; ^2^ Department of Renewable Resources, University of Alberta, Edmonton, AB, Canada

**Keywords:** Conifers, tree improvement, phenotypic variation, domestication, selection, gas exchange, phytohormones

## Abstract

Tree improvement programs select genotypes for faster growth, at both early and late stages, to increase yields over unimproved material, and the improvement is frequently attributed to genetic control in growth parameters among genotypes. Underutilized genetic variability among genotypes also has the potential to ensure future gains are possible. However, the genetic variation in growth, physiology and hormone control among genotypes generated from different breeding strategies has not been well characterized in conifers. We assessed growth, biomass, gas exchange, gene expression and hormone levels in white spruce seedlings obtained from three different breeding strategies (controlled crosses, polymix pollination, open pollination) using parents grafted into a clonal seed orchard in Alberta, Canada. A pedigree-based best linear unbiased prediction (ABLUP) mixed model was implemented to quantify variability and narrow-sense heritability for target traits. The levels of several hormones and expression of gibberellin-related genes in apical internodes were also determined. Over the first two years of development, the estimated heritabilities for height, volume, total dry biomass, above ground dry biomass, root:shoot ratio and root length, varied between 0.10 and 0.21, with height having the highest value. The ABLUP values showed large genetic variability in growth and physiology traits both between families from different breeding strategies, and within families. The principal component analysis showed that developmental and hormonal traits explained 44.2% and 29.4% of the total phenotypic variation between the three different breeding strategies and two growth groups. In general, controlled crosses from the fast growth group showed the best apical growth, with more accumulation of indole-3-acetic acid, abscisic acid, phaseic acid, and a 4-fold greater gene expression of *PgGA3ox1* in genotypes from controlled crosses versus those from open pollination. However, in some cases, open pollination from the fast and slow growth groups showed the best root development, higher water use efficiency (iWUE and δ^13^C) and more accumulation of zeatin and isopentenyladenosine. In conclusion, tree domestication can lead to trade-offs between growth, carbon allocation, photosynthesis, hormone levels and gene expression, and we encourage the use of this phenotypic variation identified in improved and unimproved trees to advance white spruce tree improvement programs.

## Introduction

White spruce (*Picea glauca* (Moench) Voss) is one of the most widely distributed tree species in Canada, and with tree improvement programs across the country (Galeano et al., 2021). In Alberta, Canada, improvement programs for white spruce mainly use open-pollinated material, however, this strategy is not sufficient to explore the full potential of productivity of white spruce plantations (Flewelling, 2008; Sklar, 2012; FGRMS, 2016; Government of Alberta, 2018; Galeano et al., 2021). In order to advance improvement in white spruce, the adoption of controlled crosses needs to be considered as it typically results in better productivity than open pollination in most cases, and this approach has been the backbone of plant domestication (Leakey, 2017a). Forest tree domestication is defined as the production of individual genotypes or clones exhibiting traits that are desirable to foresters through an active and conscious process of selection, testing, and breeding, becoming increasingly important in supplying timber, fiber, fuel, food, resistance to pests/diseases and to abiotic stressors for future generations (Pearsall, 2008; Harfouche et al., 2012; Gepts, 2014; Leakey, 2014; Leakey, 2017a). Tree domestication also has a key role to play in the mitigation of climate change and conservation of natural resources (Leakey, 2017b). The first plant breeding occurred with the domestication of wild wheat, barley, and lentils around 13,000-10,000 years ago, but the first successful selections and crosses were made with cereals in the Fertile Crescent in Eurasia (today known as Iraq, Iran, Turkey, Lebanon, and Israel) between 9,000 and 7,000 BCE (Pearsall, 2008; Abbo and Gopher, 2020). Tree domestication was first reported in date palm through hand pollination, in approximately 700 BCE (Singh et al., 2021).

Taking advantage of polymix pollination and controlled crosses, uncovers hidden variation not visible using open pollination, and can thereby maximize the benefits possible from the better trees in a population (Kanowski and Borralho, 2004). The genetic variation of trees within genotypes resulting from different breeding strategies is unknown, and trees or families with outstanding performance are commonly not included with an open pollination strategy. Genetic variation with substantial ranges has been observed, for juvenile morphological and physiological traits of spruce and pine seedlings, between seed orchards, regions, families, somatic clones, provenances and among trees within the same family (Li et al., 1993; Rweyongeza et al., 2005; Carles et al., 2012; Quesada et al., 2017; Prud’Homme et al., 2018). The main traits of interest in tree breeding programs usually involve height, diameter, wood properties, and disease and insect resistance, which are considered quantitative traits and influenced by the action of multiple genes and the environment, but undoubtedly, other traits of increasing interest in forestry are biomass, photosynthesis, water use efficiency, and δ^13^C in particular, with the increased impact of climate change (Quesada et al., 2017). Genetic variation contributes to phenotypic plasticity and, therefore, constitutes an essential factor in the adaptive capacity of trees and subsequent forest productivity (Grattapaglia et al., 2009; Verta et al., 2013; Plomion et al., 2016). Conifers have shown considerable capacity for rapid local adaptation even with their long lifespans and extensive gene flow due to wind pollination (Six et al., 2021). Furthermore, growth performance and architectural patterns of conifers established in the early stages are indicative of how their development will be, decades later, when the plant has increased substantially in size (Raj et al., 2006). Different patterns in gene expression and hormone levels (e.g. auxins, cytokinins, abscisic and gibberellic acid) allow for contrasting performance of growth and physiology of trees within families, contributing to functional innovations, genetic variation and may facilitate adaptation following environmental change (Verta et al., 2013; Park et al., 2014).

Auxins function primarily in stem elongation by promoting cell growth, and indole-3-acetic acid (IAA) is the major naturally occurring auxin (Dilworth et al., 2017). Cytokinins promote cell division and differentiation, budbreak, increase tolerance to drought stress, and inhibit cell elongation; the most naturally occurring cytokinins are trans-zeatin (tZ), dihydrozeatin (dhZ) and isopentenyladenosine (iPR) (Persson et al., 1994; Cline and Harrington, 2007; Lulsdorf et al., 2013; Dilworth et al., 2017). Abscisic acid (ABA) prevents the activation of axillary buds, inhibits elongation of internodes and seed germination, and it is responsible for the plant response to abiotic stresses (Arney and Mitchell, 1969; Dilworth et al., 2017; Shu et al., 2018). Gibberellic acid (GA) promotes stem elongation, flowering, leaf expansion, and seed germination (Dilworth et al., 2017; Shu et al., 2018). Many hormones are present in plants and plant developmental processes are controlled by complex interactions. For example, the cross-talk between auxin and cytokinin controls the apical dominance and functions mainly in the newly formed buds of the current year’s growth, while the cross-talk between ABA and GA antagonistically regulates root initiation, stem and hypocotyl elongation, and the interaction between auxins and GA regulates plant growth (Cline and Harrington, 2007; Shu et al., 2018; Akhtar et al., 2020). Furthermore, previous studies have found differences in hormone levels and GA-related genes between individuals and families of spruce and pine open-pollinated plant material, identifying these hormones as potential biomarkers for growth in trees (Kayal et al., 2011; Park et al., 2014; Galeano and Thomas, 2020).

Despite the availability of different breeding strategies, open pollination is still the dominant practice in Alberta’s tree improvement programs. We believe there is a need for genetic and phenotypic data to inform our understanding of the potential of the genetic variability when using different breeding strategies to support selection of material and genotypes and increase the genetic gain. So far, there are no studies characterizing the genetic variation of growth and physiological traits in white spruce individuals coming from different breeding strategies. Also, there are no studies analyzing the influence and changes of gene expression and hormones in spruce trees from different breeding strategies, and use of these tools in conifer tree improvement. The objectives of the present study are to: (a) evaluate the genetic variation in growth and physiology among families from three different breeding strategies (i.e. open pollination, polymix pollination, controlled cross pollination), (b) analyze the interactions between growth, physiology, hormones, and gibberellin-related genes between breeding strategies and growth groups (fast and slow).

## Materials and methods

### Selection of genotypes for breeding

This study was conducted using seeds from the G1 clonal seed orchard, as part of the G1 white spruce Controlled Parentage Program (CPP) that began in 1979 (John, 2011). The G1 clonal seed orchard is located near Grande Prairie, Alberta, Canada (lat. 55°03’51” N, long. 119°16’24” W, 720 elevation) and designed to produce seed for the G1 region in north-west central Alberta (FGRMS, 2016).The G1 clonal seed orchard was established from ramets, or grafts, collected from parent trees located in wild stands between 1988-2005, and consisted of 151 ‘founders’ (with ~12 ramets per selected parent tree (clone)). Based on the growth measurements from four progeny trials, the orchard manager ranked genotypes based on the breeding values for height (%) at a rotation age of 80, and based on the rankings, conducted three roguing. The first roguing removed six genotypes (all ramets) in fall of 2009, the second roguing removed eight genotypes (all ramets) in spring of 2010, and the third roguing removed 84 genotypes in spring of 2018 (**Supplementary Material 1A**), leaving 53 founder genotypes considered as “superior” eliminating 65% of the original 151 genotypes in the orchard by summer 2018 (John, 2011). From the 53 remaining genotypes, we selected six genotypes as females and nine different genotypes as males (**Supplementary Material 1B**), to perform and compare three different breeding strategies: controlled cross pollination (CC), polymix pollination (PM), and open pollination (OP).

### Controlled cross pollination, polymix pollination and open pollination

During summer 2018, we performed a polymix pollination (PM) and controlled cross pollination (CC), and collected seeds from open pollination (OP), with the same females used for all three breeding strategies. Controlled crosses were performed using a disconnected factorial design (Isik et al., 2017) with 15 elite parents grouped into three different clusters based on breeding values for tree height obtained from two progeny trials at ages 14 and 31. Each of the six females were also pollinated using a mix of pollen (polymix) from the nine selected males, and open-pollinated cones which were also collected from the same female trees for comparison. Male and female individuals were inspected for phenological stages of reproductive structures (strobili) in the first week of May 2018 (**Supplementary Material 2**). Reciprocal crosses were not performed. Branches selected for male strobili were marked for pollen collection. Female branches selected were covered with pollination bags (PBS 3D.75, 158mm wide x 750mm tall x 158mm deep with a UV stable vinyl window 100mm x 250mm on front, non-woven polyester, PBS International, Scarborough, United Kingdom) on May 10, 2018, prior to pollen release based on monitoring, and based on the female conelets not being receptive yet. The PBS 3D.75 bags were chosen based on Heine et al. (2020), which showed that PBS bags were more suitable than kraft bags for conelet survival when breeding loblolly pine trees. The bags were sealed with plastic ties, and sanitary pads were placed in between the bags and branches where the ties were placed to avoid damage to the branch and prevent pollen contamination (**Supplementary Material 2**). Approximately 50 male conelets were collected on May 17, 2018 from each of the nine selected clones. Next, male conelets were placed into a 20 cm diameter industrial funnel (Scepter Canada Inc., Scarborough, Canada) which allowed for separation of the pollen from the conelet and sieved through a 1.18 mm sieve (Royal Selangor Co). An equal portion of pollen (~ 1 g) from each male was combined and used for the PM. Either individually or in the pollen mix, pollen was placed into individual long nozzle squeeze bottles with a 13G size purple blunt needle (2.4 mm outer diameter x 1.8 mm inner diameter x 0.3 wall thickness) affixed to the tip. For the controlled crosses, one bottle was assigned for each individual pollen source. Seven days after bagging the branches with female conelets, pollen was applied directly to the 1-10 conelets inside each pollination bag (May 17-18 2018), by puncturing the pollination bags with the needle. This process was repeated every other day for a total of three applications to ensure each female conelet was exposed to the pollen during the receptive period. The puncture holes were resealed with black electrician’s tape and the bags remained on the branches for four weeks during fertilization (Bonner et al., 2009). The bags were removed by mid-June 2018 after conelets were no longer receptive, and the conelets continued to develop on the branches with periodic examinations through June and July 2018. Two OP cones were cut from different trees every week to evaluate maturity. All cones used in this study were collected on August 13-14, 2018 (**Supplementary Material 2**). Twenty-seven individual seedlots were collected from the CC and nine seedlots from the PM (without replication) with a range of 1-60 cones per seedlot. In addition, approximately 20 open-pollinated cones were randomly selected from each of the nine females used in the controlled crosses, representing the OP seedlots. Cones were placed in metal trays to dry for two weeks with intermittent stirring (Gärtner et al., 2011) at the Thomas Lab, University of Alberta. The dried, opened cones were taken to the Alberta Tree Improvement and Seed Center in Smoky Lake, AB, Canada, for seed extraction. Seed extraction and de-winging of seeds were done manually during September 2018 (**Supplementary Material 2**). The extracted seeds were sorted in two steps, first by size, through a Canadian Standard Sieve size 16 (1.18 mm) (W.S. Tyler Company of Canada Ltd., St. Catharines, Ontario, Canada) to remove the smallest seeds which were discarded. Then, wings and waste were subsequently removed using a size 8 (2.36 mm) sieve. Second, the seeds were sorted by weight through a series of blowers to remove empty and aborted seeds (**Supplementary Material 2**). Extracted seeds were placed into 10 x 15 cm metal bags metallic bags (ULINE, Milton, Ontario, Canada) and stored in a -20°C freezer until grown.

### Experimental design and condition of plants at the greenhouse

The experiment was a randomized complete block design (**Supplementary Material 3**) with a total of 18 families, 10 blocks and three trees/family/block. To assess the first objective (evaluate the genetic variation of growth and physiology among families from three different breeding strategies), we categorized all genotypes into two breeding value rankings based on height (%): mid BV (2.5 to 6.0), high BV (6.0 to 12) (**Supplementary Material 1**). To assess the second objective (analyze the interactions between growth, physiology, hormones, and gibberellin-related genes with the breeding strategies and growth groups), we took the two families with the lowest BVs from the mid BV ranking (genotypes 193 and 122, BV=3.9), and we called them the “slow growth” group (**Table 1**; **Supplementary Material 1**).We then took the two families with the highest BVs from the high BV ranking (genotypes 927 and 138, BV=8.9), and we called them the “fast growth” group (**Table 1**; **Supplementary Material 1**). Seed was removed from the -20°C freezer and pre-treated at 4°C in the fridge for two weeks, after which a total of 180 seeds were sown (10 per family, each in 10 blocks, 18 families) on January 16, 2019 at a commercial forest nursery (Bonnyville, Alberta, Canada). Seedlings began emerging on February 1, 2019 and were grown until November 29, 2019 (10 months), and subsequently lifted, packed and stored at -2°C on December 1, 2019 at Bonnyville, Alberta, Canada. On January 20, 2020 seedlings were removed from cold storage and planted into 2L pots filled with Sunshine Mix #4 (Sungro, Vilna, Alberta, Canada) and grown at the Biological Sciences greenhouse, University of Alberta, for their second growing season, between January 28 and April 28, 2020 (Day 92 of the experiment). Plants were grown under natural light supplemented by cool-white fluorescent lamps (400 µmol m^-2^ s^-1^) and provided with a 16/8 hour photoperiod and maximum day and night temperatures of 25°C and 18°C, respectively. Seedlings were irrigated three times per week, fertilized using ‘pH Reducer’ fertilizer (Plant-Prod Solutions Inc, Brampton, Canada) and Iron Chelate/Rexolin (FeEDTA 13.3%) (Yara Company, Regina, Canada) once per week, and fans were used to ensure good air circulation throughout the greenhouse. On April 28, 2020 (Day 92), seedlings were measured and harvested including internodes, stem (RNA extraction and hormone analysis), needles, branches and roots.

**Table 1:** Experimental design of the study, with a total of six genotypes used as females, six breeding value (BV) groups, two growth groups (slow growth as the average of F193 and F122; fast growth as the average of F927 and F138), three breeding strategies [the same female was used for OP (open pollination), PM (polymix pollination), CC (controlled crosses)]. The breeding value of each genotype used as female and male is also included.

Genotypes used as Female (BV)*	BV group	Growth group	Breeding strategies		
			OP**	PM (BV)***	CC [Female X Male (BV)]
**F193** (3.93)	Mid BV	Slow growth	**F193** X (53 males + external pollen)	**F193** X 9 males (7.27)	**F193** X M1045 (7.44)
**F122** (3.94)	Mid BV	Slow growth	**F122** X (53 males + external pollen)	**F122** X 9 males (7.27)	**F122** X M1047 (8.26)
**F754** (5.63)	Mid BV	–	**F754** X (53 males + external pollen)	**F754** X 9 males (7.27)	**F754** X M966 (9.49)
**F129** (7.89)	High BV	–	**F129** X (53 males + external pollen)	**F129** X 9 males (7.27)	**F129** X M756 (6.31)
**F927** (8.87)	High BV	Fast growth	**F927** X (53 males + external pollen)	**F927** X 9 males (7.27)	**F927** X M991 (6.49)
**F138** (8.91)	High BV	Fast growth	**F138** X (53 males + external pollen)	**F138** X 9 males (7.27)	**F138** X M752 (6.70)

*The females (bold) are overlapped between the breeding strategies. **Open pollination includes the 53 genotypes in the G1 orchard and external pollen from other sources (orchards and natural stands). ***Genotypes contributing as males for polymix: 199, 756, 991, 752, 115, 1045, 1002, 1047, 966 (Average BV=7.27).

### Measurements of growth, biomass, and gas exchange parameters

Final height was measured with a meter stick and diameter was measured with a digital caliper, for all seedlings (n=180), at Day 92. Volume was calculated with the equation V=π*(D/2)^2^*H at each of the five measurement points, following Galeano and Thomas (2020). Apical internode length was measured to the nearest 0.1 mm at Day 92 of the experiment. Above ground tissue, which includes branches, needles and stem, was collected and placed into paper bags. Roots were stored at -20°C for two months prior to being thawed and carefully washed. Subsequently, root length was measured to the nearest 0.1 mm and placed back into paper bags. All tissue (above ground tissue and roots) was dried for 48 hours at 60°C, and dry weights were measured using a digital scale, model AV53 (readability 0.001 g, OHAUS Adventurer Pro, Melrose, MA, USA). Gas exchange parameters were measured on all seedlings (n=180) with a CIRAS-3 Portable Photosynthesis System (PP systems, Amesbury, USA) using the conifer cuvette at Day 92 of the experiment. Measurements were taken on needles from the top branch to ensure a uniform phenological stage for all plants. Four traits were measured: photosynthesis (A; µmol CO_2_ m^-2^ s^-1^), transpiration (E; mmol H_2_0 m^-2^ s^-1^), stomatal conductance (gs; mol H_2_0 m^-2^ s^-1^), and intrinsic water use efficiency (iWUE=A/gs; µmol CO_2_ mol^-1^ H_2_0). After the measurements were concluded, the small branch with needles, used for the gas exchange measurements, was cut off and frozen in liquid nitrogen. The projected needle surface area of the needles (cm^2^) was calculated using WinSEEDLE software, version 2004 (Regent Instruments, Quebec, Canada) to correct the gas exchange measurements.

### Harvesting of needles for δ^13^C analysis

On April 28, 2020 (Day 92), a subsample of 36 trees from three blocks were chosen for harvesting of needles for δ^13^C analysis, corresponding to the genotypes 193 and 122, (lowest BV=3.9, both families with same value) and genotypes 927 and 138, (highest BV=8.9, both families with same value) from the three breeding strategies, for a total of 12 families (see Experimental Design above). A total of 100 mg of needles from the top branch were harvested into small paper bags, dried for 72 hours at 65°C, and ground using 25 mL stainless steel metal jars, 20 mm metal balls, with a Qiagen TissueLyser II (Qiagen, Redwood City, CA, USA). Approximately 50 mg of ground sample per tree was sent to the Stable Isotope Lab of InnoTech Alberta (Victoria, BC, Canada) for δ^13^C analysis. Samples were analyzed using an established method on a MAT 253 mass spectrometer with Conflo IV interface (Thermo Fisher Scientific, Waltham, MA, USA.), and a Fisons NA1500 EA (Fisons Instruments, Milano, Italy), providing a bulk analysis of carbon discrimination (δ^13^C). In brief, approximately 1.0 mg of solid sample was weighed into tin capsules and then dropped into a combustion reactor that produces CO_2_. The separated CO_2_ was then transferred to the mass spectrometer for isotopic measurement. Multiple in-house standards, calibrated relative to international standards, were also run as samples to allow the results to be normalized and reported vs Vienna Pee Dee Belemnite (VPDB) (δ^13^C).

### Harvesting of the apical internode

The same subsample of 36 trees, from three blocks chosen for δ^13^C, were used for harvesting the apical internode. After manually removing all needles, we used a scalpel to cut and collect at least 1.0 g of the apical internode. The scalpel was cleaned with 50% ethanol and rinsed with distilled water between samples. After cutting, the samples were wrapped in a double-layer of aluminum foil, labelled and immediately frozen by immersion in liquid nitrogen at the greenhouse and stored at -80°C prior to grinding for RNA extraction and hormone analysis (**Supplementary Material 4**). Two weeks after storing the material at -80°C, the 36 samples were ground into a fine powder using liquid nitrogen with a mortar and pestle, and the sample was divided into two Eppendorf tubes of 1.5 ml, each with 0.5 g of ground apical internode, for the RNA extraction and hormone analysis.

### RNA extraction, cDNA synthesis, and expression of GA-related genes

Using 0.5 g of ground apical internode, total RNA was extracted using the ‘Purelink RNA Mini Kit’, ‘Plant RNA Isolation Aid’ and ‘PureLink DNAse’ (Thermo Scientific Inc, Waltham, USA), following the manufacturer’s instructions. RNA extractions were performed in the Karst Lab, University of Alberta. A nanoDrop ND-1000 spectrophotometer (Thermo Scientific Inc, Waltham, USA) was used to check the RNA quality and concentration. The cDNA synthesis was performed using a total of 0.5 μg RNA from each sample, with the Superscript III reverse transcriptase (Thermo Scientific Inc, Waltham, USA). The *PgGA3ox1, PgGA20ox1, PgDELLA1* and *PgGID1* genes were selected based on previous studies (Kayal et al., 2011; Galeano and Thomas, 2020). Also, we used gene-specific primers for the target and reference genes based on previous work (Galeano and Thomas, 2020) (**Supplementary Material 5**) and determined the standard curve with several cDNA dilutions and the melting curve (**Supplementary Material 6**). Quantitative reverse transcription PCR (qRT-PCR) was performed for the four target GA-related genes of white spruce (*PgGA3ox1, PgGA20ox1, PgDELLA1, PgGID1*) using the 36 samples from the apical internode. PCR reactions were performed in 10 mL, containing SYBR Green master mix [0.2 mM dNTPs, 0.3 U Platinum Taq Polymerase (Invitrogen, Waltham, USA), 0.25X SYBR Green, and 0.1X ROX], 50 ng of cDNA and 300 nM of each primer. Three technical replicates for each reaction were analyzed on an ABI PRISM 7900HT Sequence Detection System (Applied Biosystems, Waltham, USA) where the first step was 95°C for 2 min followed by 40 cycles of 95°C for 15 s and 60°C for 1 min. The qRT-PCR experiments were done at the Molecular Biology Service Unit, University of Alberta. Melting curves were generated using the following program: 95°C for 15 s, 60°C for 15 s, and 95°C for 15 s. Translation initiation factor 5A (*PgTIF5A*, GenBank DR448953) was used as a reference gene, following previous work (Kayal et al., 2011; Galeano and Thomas, 2020). Two variables were used from the gene expression: absolute and relative expression. Absolute transcript levels were quantified using standard curves for the target genes (*PgGA3ox1, PgGA20ox1, PgDELLA1, PgGID1*) and reference gene (*PgTIF5A*) for each tissue per family per treatment (Kayal et al., 2011; Galeano and Thomas, 2020), and these values were used for the fixed-effect mixed model, interaction plots and correlations. Relative expression was calculated following the double delta C_T_ method (Rao et al., 2013), using the control plants as a calibrator to normalize the values between different plates, and the reference gene (*PgTIF5A*) as the control gene.

### Hormone analysis

A total of 0.5 g of ground apical internode from the 36 samples were sent to the Aquatic and Crop Resource Development Research Center, National Research Council of Canada (NRCC), Saskatoon, Saskatchewan, Canada, for the quantification of abscisic acid (ABA), abscisic acid glucose ester (ABA-GE), 7’-hydroxy-abscisic acid (7’OH-ABA), phaseic acid (PA), indole-3-acetic acid (IAA), zeatin-O-glucoside-trans (ZOG-t), zeatin-O-glucoside-cis (ZOG-c), zeatin riboside-trans (ZR-t), dihydrozeatin riboside (dhZR), isopentenyladenosine (iPR), and gibberellin 3 (GA3). For the calibration curves, ABA-GE, PA and 7’- OH-ABA were synthesized and prepared at NRCC, Saskatoon, Canada. Also, ABA, IAA, ZR-t, iPR, were purchased from Sigma–Aldrich (Burlington, MA, United States), and ZOG-t, ZOG-c, dhZR and GA3 were purchased from OlChemim Ltd. (Olomouc, Czech Republic). Deuterated forms of the hormones were used as internal standards: d4-ABA, d5-ABA-GE, d4-7’-OH-ABA, d3-PA were synthesized and prepared at NRCC, Saskatoon, Canada (Abrams et al., 2003; Zaharia et al., 2005),, d5-IAA was purchased from Cambridge Isotope Laboratories (Andover, MA), d5-ZOG-t, d5-ZOG-c, d3-ZR-t, d3-dhZR, d6-iPR, and d2-GA3 were purchased from OlChemim Ltd. (Olomouc, Czech Republic). Analysis was performed on a UPLC/ESI-MS/MS utilizing a Waters ACQUITY UPLC system, equipped with a binary solvent delivery manager and a sample manager coupled to a Waters Micromass Quattro Premier XE quadrupole tandem mass spectrometer *via* a Z-spray interface. The MassLynx™ and QuanLynx™ (Micromass, Manchester, UK) were used for data acquisition and data analysis. The procedure for quantification of ABA and ABA catabolites, cytokinins, auxins, and gibberellins in plant tissue was performed using a modified procedure described in previous work (Lulsdorf et al., 2013). Briefly, the analyses utilize the Multiple Reaction Monitoring (MRM) function of the MassLynx v4.1 (Waters Inc) control software. The resulting chromatographic traces are quantified off-line by the QuanLynx v4.1 software (Waters Inc.) wherein each trace is integrated and the resulting ratio of signals (non-deuterated/internal standard) is compared with a previously constructed calibration curve to yield the amount of analyte present (ng per sample). Calibration curves were generated from the MRM signals obtained from standard solutions based on the ratio of the chromatographic peak area for each analyte to that of the corresponding internal standard. The QC samples, internal standard blanks and solvent blanks were also prepared and analyzed along with each batch of tissue samples.

### Statistical analysis and models

Assumptions of homogeneity of variance and deviation of residuals from the normal distribution were confirmed before proceeding with the ANOVAs for the different models and mean comparisons. First, we ran a family genetic model (Isik et al., 2017) (Eq. 1), to calculate variance components for each parameter, heritabilities and BLUPs per family:


(Eq. 1) $$y=X\beta +Z_{1}BS+Z_{2}Fam+e$$

Where *β* is the vector of the block fixed effect; *BS* is the vector of breeding strategy fixed effect; *Fam* is the vector of random family effects, ~N(0, Aσ^2^_F_); e is the vector of random residual effects, ~N(0, Iσ^2^_F_); A is the pedigree matrix. Narrow sense heritability (h^2^) is then estimated using the additive ( $\sigma_{a}^{2}$ ) and residual ( $\sigma_{e}^{2}$ ) variances (Fernández-Paz et al., 2021) (Eq. 2):


(Eq. 2) $$h^{2}=\sigma_{a}^{2}/\left[\sigma_{a}^{2}+\sigma_{e}^{2}\right]$$

Second, we ran a fixed-effect mixed model to assess main and interaction effects of the breeding strategies and growth groups (Eq. 3):


(Eq. 3) $$Y_{ijkl}=\text{µ}+GG_{i\;}+BS_{\;j}+Bl_{k}+{\left(GG*BS\right)}_{ij}+e_{ijkl}$$

Where *Y_ijkl_* is the measured value for each growth group, breeding strategy and block; *μ* is the overall mean; *GG_i_* is the fixed growth group effect; *BS_j_* is the fixed breeding strategy effect; *Bl_k_* is the random block effect; (GG*BS)*_ij_* is the fixed interaction effect between growth group and breeding strategy; *e_ijkl_* is the residual error. For Equation 1, a total of 180 plants were analyzed (10 blocks, 18 families). For Equation 2, a total of 36 plants were analyzed (3 blocks, 12 families) to test the extremes only. The models were assessed with α≤0.05 and α≤0.01 (as a more stringent option). Significant differences between means of all traits (growth, biomass, gas exchange, transcript and hormone levels) were determined by Tukey’s HSD test with overall α≤0.05. Pearson’s correlation (r) coefficients between growth, transcript and hormone levels were estimated with α≤0.05 and α≤0.01. All statistical analyses were carried out using the R environment, using the ASReml package (Gilmour et al., 2009). Graphics were generated using the ggplot2 package (Wickham, 2016) in the R environment and Excel.

## Results

### Genetic variation of growth and physiology of white spruce seedlings among different breeding strategies

**Figure 1:**
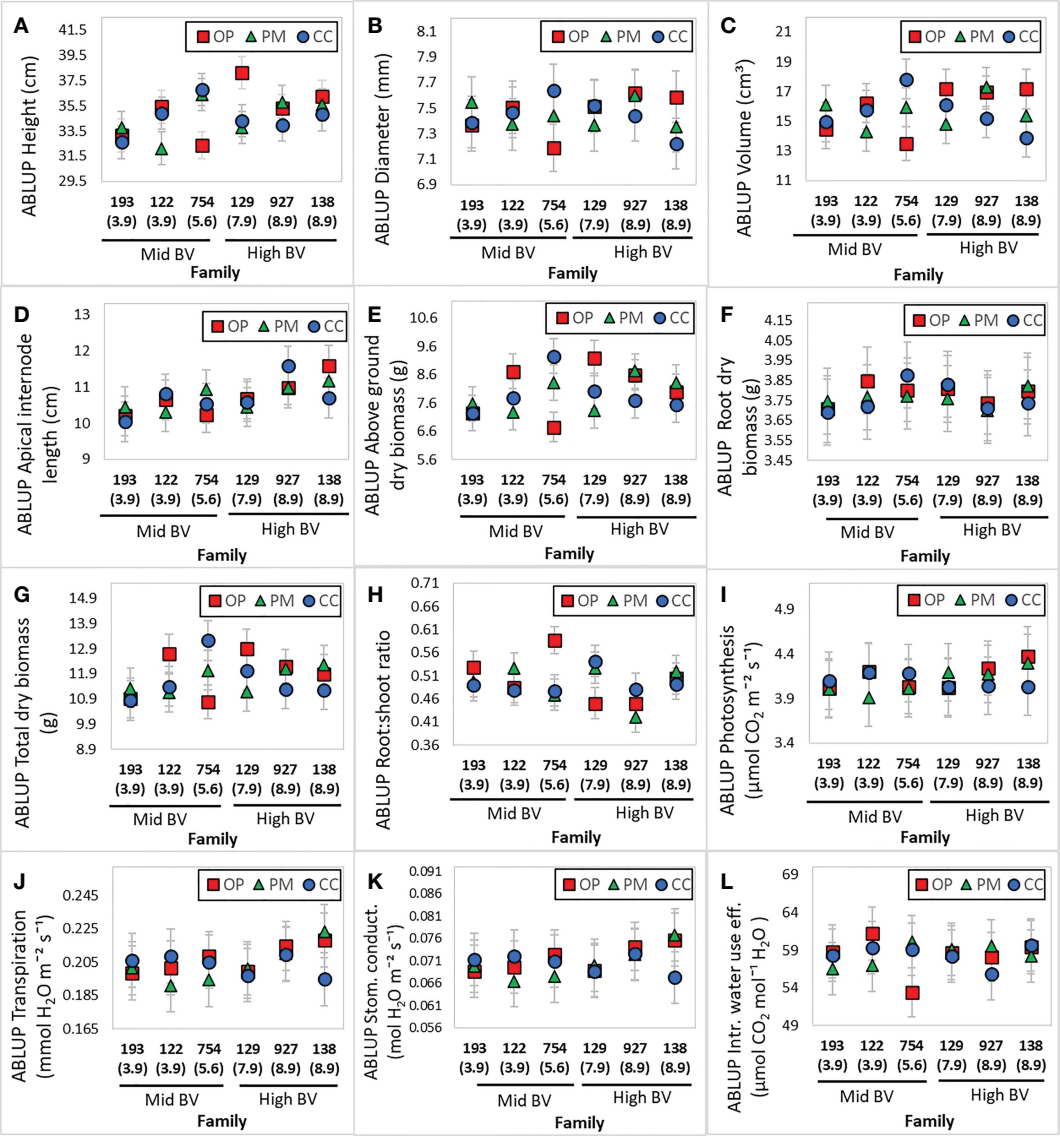
Best linear unbiased predictions (BLUPs) of the ‘family’ random effect from the ‘Family Genetic Model’ for growth, biomass and gas exchange parameters (n=180, 10 blocks, 18 families) in 2-year-old white spruce seedlings. **(A)** Height, **(B)** Diameter, **(C)** Volume, **(D)** Apical internode length, **(E)** Above ground dry biomass, **(F)** Root dry biomass, **(G)** Total dry biomass, **(H)** Root:shoot ratio, **(I)** Photosynthesis, **(J)** Transpiration, **(K)** Stomatal conductance, **(L)** intrinsic water use efficiency. For the model, we used the pedigree matrix (also called A matrix) with the different relationships between half- and full- sibs among the three different breeding strategies: OP (open pollination), PM (polymix pollination), CC (controlled crosses). Each figure includes the mid and high BV groups. Families were ordered from the lowest to the highest BV (value under each family). Last measurements and harvesting were done on Day 92 (28 April 2020).

A total of 18 families were obtained from the three breeding strategies studied: open pollination (OP), polymix pollination (PM) and controlled cross pollination (CC). Means of each of the two different breeding values and three different breeding strategies are shown in **Table 2**. The highest *h^2^* was found for height (0.21), followed by above ground dry biomass (0.16), and root:shoot ratio (0.14) (**Table 3**). All dry biomass, volume and root length showed lower heritabilities of 0.12, 0.11, 0.10, respectively (**Table 3**). Diameter, apical internode length, root dry biomass and gas exchange parameters showed a heritability below 0.08 (**Table 3**). We obtained the Best Linear Unbiased Predictions (BLUPs) using the A matrix for each of the 18 families with overlapping ‘females’ using Model 1 (see Materials and Methods). We expected that families in the High BV ranking would perform better than those in the Mid BV ranking, but in general, families from both rankings showed no clear tendency in growth, biomass or gas exchange parameters for any breeding strategy (**Figure 1**). Families 129 and 138 (High BV) coming from the PM and CC showed smaller ABLUPS for height, diameter and volume compared to families 122 and 754 coming from OP (**Figures 1A–C**). Family 927 (High BV) showed the highest ABLUPs for apical internode length among all families and breeding strategies, but family 138 (High BV), coming from CC, showed similar ABLUP values compared to families 122 and 754 (Mid BV), coming from CC, PM and OP (**Figure 1D**). Families 129, 927 and 138 (High BV) showed the lowest ABLUPs for above ground dry biomass, root dry biomass and total dry biomass compared to family 754 (Mid BV) (**Figures 1E–G**). On the other hand, CC and PM performed better than OP for family 754 for height, diameter, volume, apical internode length, above ground biomass, and total dry biomass (**Figures 1A–G**). For root:shoot ratio, OP showed a higher ABLUP for family 754 than the rest of the families (**Figure 1H**). Regarding gas exchange parameters, ABLUPs showed smaller differences between families compared to growth and biomass traits. For photosynthesis, transpiration and stomatal conductance, family 138 (High BV) showed lower values compared to the other families, and families 754 (Mid BV) and 927 (High BV) showed OP and CC with almost the same ABLUPs (**Figures 1I–K**). Families 129 and 138 (High BV) showed the same iWUE for all breeding groups (**Figure 1L**). In some cases, CC showed the highest ABLUPs for diameter, volume, root dry biomass, and total dry biomass (family 754), and PM showed the highest ABLUPs for transpiration and stomatal conductance (family 138). In other cases, OP showed the highest ABLUPs for height (family 129), apical internode length and photosynthesis (family 138), root:shoot ratio (family 754), iWUE (family 122).

**Table 2:** Means (± SE) for traits including growth, biomass, gas exchange parameters (n=180, 10 blocks, 18 families), δ^13^C, hormone levels, absolute gene expression parameters (n=36, 3 blocks, 12 families) in 2-year-old white spruce seedlings from three different breeding strategies (OP, open pollination; PM, polymix pollination; CC, controlled crosses) and two different levels of breeding values (BV) (medium and high).

Traits ^1^	Breeding strategy (mean ± SE)					
	OP		PM		CC	
	Mid BV	High BV	Mid BV	High BV	Mid BV	High BV
H	32.6 ± 4.4	37 ± 4.2	33.9 ± 5.3	35.2 ± 4.5	34.7 ± 4.9	34.1 ± 3.7
D	7.2 ± 0.9	7.7 ± 1.0	7.3 ± 1.0	7.2 ± 1.3	7.8 ± 1.3	7.4 ± 1.3
Vol	13.7 ± 4.6	17.3 ± 5.1	14.5 ± 4.5	15.2 ± 6.0	17.3 ± 6.5	15.2 ± 5.7
Ap.Int.L	9.6 ± 2.2	11.2 ± 2.5	10.2 ± 2.7	10.9 ± 2.1	10.4 ± 2.6	11.5 ± 3.2
Root L	29.4 ± 7.	27.4 ± 3.5	25.3 ± 4.8	29.8 ± 8.5	27.2 ± 5.2	27.5 ± 4.3
Ab. g. dm	7.1 ± 3.0	8.6 ± 2.4	7.8 ± 1.9	8.5 ± 2.9	8 ± 2.6	7.5 ± 1.8
Root dm	3.8 ± 1.3	3.9 ± 0.8	3.7 ± 1.1	3.7 ± 1.2	3.8 ± 1.4	3.8 ± 1.0
Total dm	11 ± 4.1	12.4 ± 2.9	11.5 ± 2.7	12.1 ± 3.6	11.8 ± 3.7	11.2 ± 2.4
R:S ratio	0.58 ± 0.2	0.48 ± 0.1	0.48 ± 0.1	0.47 ± 0.2	0.48 ± 0.1	0.52 ± 0.1
A	3.7 ± 2.0	4.5 ± 2.2	3.2 ± 1.9	4.7 ± 2.4	4.6 ± 2.7	3.9 ± 2.1
E	0.17 ± 0.1	0.21 ± 0.1	0.17 ± 0.1	0.23 ± 0.1	0.23 ± 0.1	0.2 ± 0.1
gs	0.06 ± 0.02	0.07 ± 0.04	0.06 ± 0.03	0.08 ± 0.04	0.08 ± 0.04	0.07 ± 0.03
iWUE	59.4 ± 34.1	64.5 ± 13.6	54.3 ± 32.2	58.7 ± 17.1	59.7 ± 16.0	55.3 ± 19.6
δ^13^C	-29.6 ± 1.3	-29 ± 0.9	-28.8 ± 0.9	-28.7 ± 0.9	-28.8 ± 1.0	-28.6 ± 0.7
ABA	7.7 ± 5.5	6.7 ± 2.5	5.7 ± 2.4	6.1 ± 1.9	6 ± 2.4	6.4 ± 3.4
ABA-GE	4.6 ± 2.8	3.8 ± 1.9	4.5 ± 2.8	5.1 ± 3.2	4.5 ± 3.4	5.9 ± 4.3
PA	23.9 ± 12.8	18.6 ± 10.7	26.1 ± 19.8	30.3 ± 10.7	22 ± 11	27.3 ± 14.8
7’OH-ABA	8.5 ± 5.4	7.6 ± 2.6	15.1 ± 17.3	11.4 ± 10.5	10.4 ± 6.1	15.2 ± 19.2
ZOG-t	4.7 ± 2.0	6.1 ± 3.0	7.6 ± 3.8	5.6 ± 3.1	5.7 ± 3.5	9.4 ± 9.2
ZOG-c	6.7 ± 2.3	8.1 ± 2.9	6.3 ± 2.4	7.8 ± 3.7	8 ± 3.2	12.1 ± 5.8
ZR-t	27.8 ± 14.2	46.1 ± 11.6	44.7 ± 24.2	35.2 ± 28.5	36.2 ± 19.7	41.5 ± 28.2
dhZR	7 ± 4.7	8.6 ± 4.1	12.3 ± 8.7	9.1 ± 8.0	8.2 ± 2.5	8.1 ± 5.0
iPR	6.9 ± 2.6	10.6 ± 4.2	10 ± 3.9	7.8 ± 2.3	7.7 ± 3.0	9.3 ± 6.2
IAA	1.1 ± 0.2	1.4 ± 0.5	1.1 ± 0.3	1.2 ± 0.3	1.1 ± 0.2	1.4 ± 0.3
GA3	2.1 ± 2.5	1.4 ± 2.0	2.4 ± 3.3	0.3 ± 0.2	0.5 ± 0.7	1.4 ± 1.6
*PgGA3ox1*	10.6 ± 1.5	9.9 ± 1.4	10 ± 0.9	9.8 ± 1.1	10.3 ± 1.2	10.8 ± 1.1
*PgGA20ox1*	8.1 ± 0.5	8.4 ± 0.8	8.1 ± 0.6	8.8 ± 0.4	8.3 ± 0.5	8.2 ± 0.5
*PgDELLA1*	5.3 ± 0.4	4.8 ± 0.9	5.4 ± 0.3	5.3 ± 0.3	5.3 ± 0.1	5.3 ± 1.2
*PgGID1*	7.7 ± 0.5	7.4 ± 0.4	7.8 ± 0.4	7.5 ± 0.3	7.8 ± 0.2	7.7 ± 0.3

^1^ H, Height (cm); D, Diameter (mm); Vol, Volume (cm^3^); Ap. Int. L, Apical Internode Length (cm); Root L, Root length (cm); Ab. g. dm, Above ground dry biomass (g); Root dm, R oot dry biomass (g); Total dm, Total dry biomass (g); R:S ratio, Root:Shoot ratio; A, photosynthesis (µmol CO_2_ m^-2^ s^-1^); E, transpiration (mmol H_2_0 m^-2^ s^-1^); gs, stomatal conductance (mol H_2_0 m^-2^ s^-1^); iWUE, intrinsic Water Use Efficiency (µmol CO_2_ mol^-1^ H_2_0); δ^13^C , δ^13^C (‰; VPDV); ABA, Abscisic acid (ng/g) (1.0e+2); ABA-GE, Abscisic acid glucose ester (ng/g) (1.0e+3); PA, Phaseic acid (ng/g); 7’OH-ABA, 7’-hydroxy-abscisic acid (ng/g); ZOG-t, Zeatin-O-glucoside-trans (ng/g); ZOG-c, Zeatin-O-glucoside-cis (ng/g); ZR-t, Zeatin riboside-trans (ng/g); dhZR, Dihydrozeatin riboside (ng/g); iPR, Isopentenyladenosine (ng/g); IAA, Indole-3-acetic acid (ng/g) (1.0e+2); GA3, Gibberellin 3 (ng/g) (1.0e+2). PgGA3ox1; PgGA20ox1; PgDELLA1 and PgGID1 are the GA-related genes analyzed in this study using their absolute transcript levels; this unit is the transcript mean of each target gene divided by the transcript mean of the reference gene (PgTIF5A) per reaction. Last measurements and harvesting were done on Day 92 (28 April 2020).

**Table 3:** Results from the ‘Family Model’ for the growth, biomass and gas exchange parameters (n=180, 10 blocks, 18 families) in 2-year-old white spruce seedlings from three different breeding strategies (OP, open pollination; PM, polymix pollination; CC, controlled crosses).

Traits	*P*-value (Breeding Strategy)	Variance Component Family	Variance component Residual	h² ( ± SE)
Height	<0.01	4.68	18.12	**0.21** (± 0.11)
Diameter	<0.01	0.05	1.16	0.04 (± 0.01)
Volume	<0.01	3.17	28.36	**0.11** (± 0.09)
Apical internode length	<0.01	0.46	6.15	0.07 (± 0.02)
Root length	<0.01	4.3	36.84	**0.10** (± 0.09)
Above ground dry biomass	<0.01	0.92	4.89	**0.16** (± 0.03)
Root dry biomass	<0.01	0.02	1.55	0.01 (± 0.01)
Root-Shoot ratio	<0.01	0.002	0.0016	**0.14** (± 0.02)
Total dry biomass	<0.01	1.15	10.09	**0.12** (± 0.03)
Photosynthesis	<0.01	0.01	0.51	0.02 (± 0.01)
Transpiration rate	<0.01	0.0003	0.008	0.04 (± 0.01)
Stomatal conductance	<0.01	0.004	0.12	0.03 (± 0.02)
Intrinsic water use efficiency	<0.01	0.013	0.45	0.03 (± 0.01)

For the genetic model, we used the pedigree matrix (A) to obtain the Best Linear Unbiased Predictions (BLUPs) for the family as random factor (see Materials and Methods, and **Figure 1**). The table includes the *P*-values for the breeding strategy (fixed factor) from the Wald-F test, the variance component of family and residual, and the narrow-sense heritability (h^2^) for each trait. Moderate narrow-sense heritabilities are indicated in bold. Last measurements and harvesting were done on Day 92 (28 April 2020).

### Genetic variation among individuals within families for growth, physiology, hormone levels and gene expression

**Figure 2:**
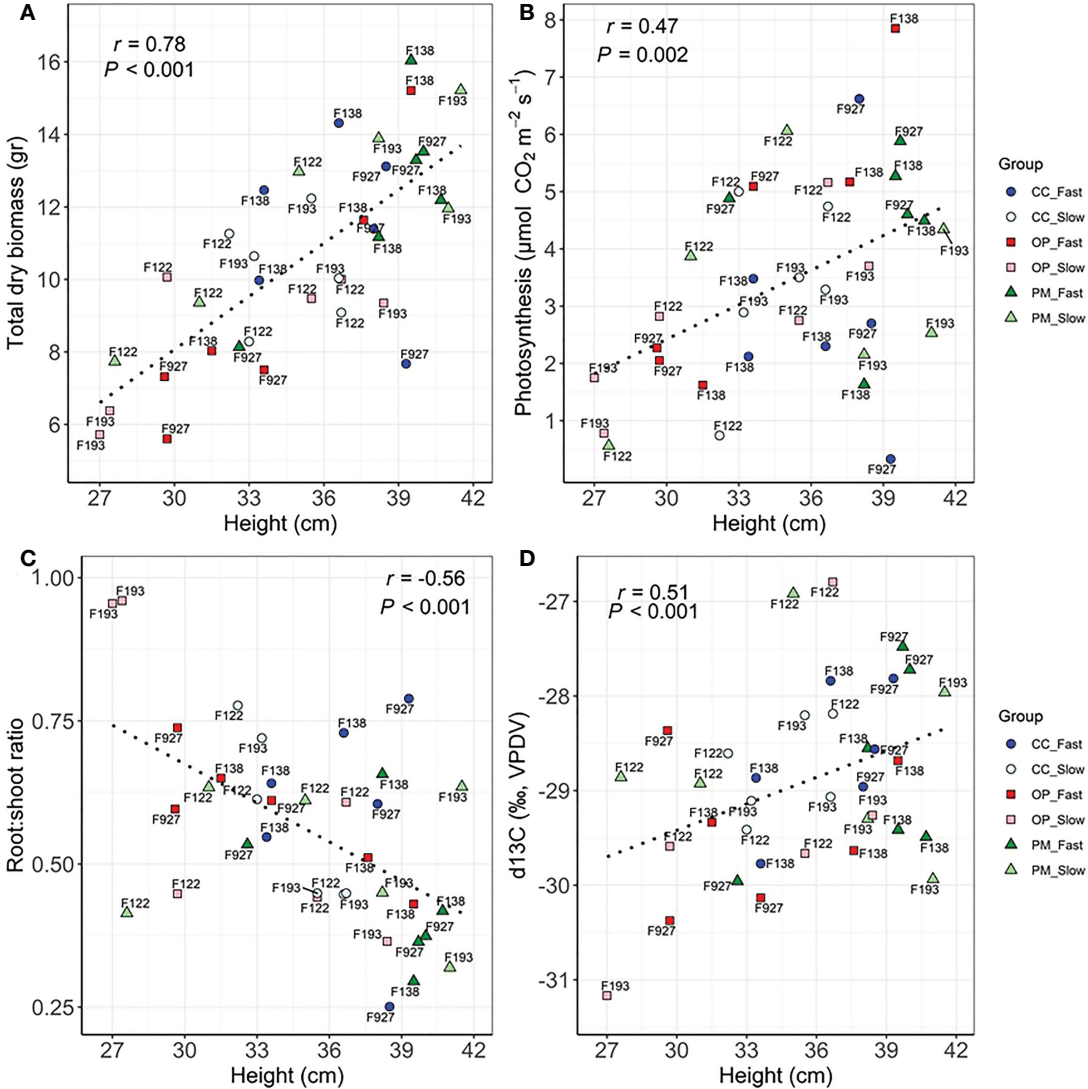
Scatterplots between **(A)** height and total dry biomass, **(B)** height and photosynthesis, **(C)** height and Root:shoot ratio, **(D)** height and δ^13^C among groups (controlled crosses (CC), polymix (PM) and open pollination (OP) for slow (F193, F122) and fast (F927, F138) growth in 2-year-old white spruce seedlings. Pearson’s correlation (r) and p-values (*P*) corresponding to each correlation are shown in each graphic. Each point corresponds to each tree (n=36, 3 blocks, 12 families) (see family number for each point in the graphic). The six groups are detailed for each family with colours and shapes. Last measurements and harvesting were done on Day 92 (28 April 2020).

**Figure 3:**
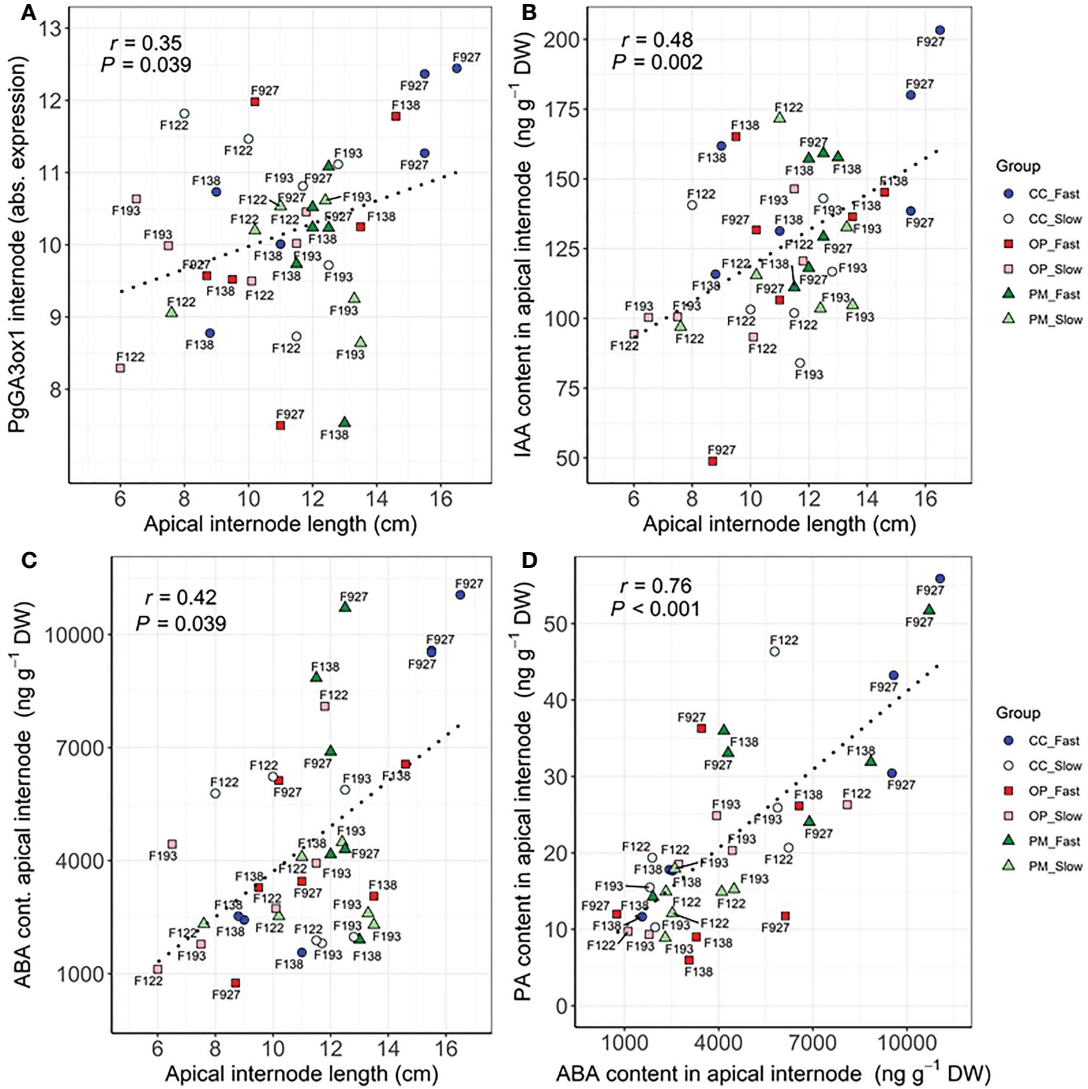
Scatterplots between **(A)** apical internode length and *PgGA3ox1* expression in apical internode, **(B)** apical internode length and IAA content in apical internode, **(C)** apical internode length and ABA content in apical internode, **(D)** ABA content and PA content in apical internode among groups (controlled crosses (CC), polymix (PM) and open pollination (OP) for slow (F193, F122) and fast (F927, F138) growth in 2-year-old white spruce seedlings. Pearson’s correlation (r) and p-values (*P*) corresponding to each correlation are shown in each graphic. Each point corresponds to each tree (n=36, 3 blocks, 12 families) (see family number for each point in the graphic). The six groups are detailed for each family with colours and shapes. Last measurements and harvesting were done on Day 92 (28 April 2020).

Significant correlations were found between growth, biomass, gas exchange, δ^13^C and hormone traits (**Table 4**). As expected, the highest correlations were found between height vs. apical internode length (r=0.82, P<0.01), height vs. total dry biomass (r=0.78, P<0.01), volume vs. total dry biomass (r=0.75, P<0.01), photosynthesis vs. transpiration (r=0.78, P<0.01), stomatal conductance vs. above ground dry biomass (r=0.62, P<0.01), *PgGA3ox1* vs. ABA (r=0.63, P<0.01), ABA vs. PA (r=0.76, P<0.01) (**Table 4**). We found significant correlations between height vs. photosynthesis (r=0.47, P<0.01), height vs. root:shoot ratio (r=-0.56, P<0.01), height vs. δ^13^C (r=0.51, P<0.001), apical internode length vs. *PgGA3ox1* (r=0.35, P<0.05), apical internode length vs. IAA (r=0.48, P<0.01), apical internode length vs. ABA (r=0.42, P<0.05), apical internode length vs. PA (r=0.76, P<0.01) (**Table 4**). Scatterplots for height vs. total dry biomass (**Figure 2A**), photosynthesis (**Figure 2B**), root:shoot ratio (**Figure 2C**), and δ^13^C (**Figure 2D**), showed considerable genetic variability among all seedlings analyzed in the three breeding strategies. The three replications for family F138 from PM showed similar values for height, but a range between 11 and 16 g for total dry biomass (**Figure 2A**), while the repetitions for family F138 from OP showed a much greater range for height, varying between 32 and 40 cm (**Figures 2A–D**). The three plants analyzed for families 122 and 193 from PM showed a similar response among them for photosynthesis, but family 927, from CC, had a range from 0.4 to 6.6 µmol CO_2_ m^-2^ s^-1^ (**Figure 2B**). Trees within families for root:shoot ratio and δ^13^C also showed contrasting values, such as family F193 from OP and 927 from CC (**Figure 2C**), family 927 from PM and F138 from CC (**Figure 2D**). In general, however, CC and PM plants showed the highest values for total dry biomass, photosynthesis, δ^13^C, and the lowest root:shoot ratios (**Figures 2A–D**). Scatterplots for apical internode length vs. *PgGA3ox1* (**Figure 3A**), IAA (**Figure 3B**), ABA (**Figure 3C**), and ABA with PA (**Figure 3D**), again showed large genetic variability, but with particular individuals outstanding in their performance at the molecular level within a given breeding strategy. For example, two individuals from F927 coming from CC excelled in apical internode growth, as well as gene expression of *PgGA3ox1* and levels of IAA, ABA, PA, and one individual from F927, coming from OP, performed poorly not only for growth but also for hormone levels (**Figures 3A–D**). In general, individuals from the different growth groups and breeding strategies showed significant overlap in hormone levels (**Figures 3A–D**).

**Table 4:** Pearson’s correlation matrix between growth, biomass and gas exchange traits for white spruce seedlings (n=36, 3 blocks, 12 families).

Trait ^+^	H	D	V	AIL	E	gs	A	iWUE	ADM	RDM	RL	R:S	TDM	δ^13^C	GA3ox	ABA	PA	ZOG	iPR	IAA
**D**	0.35*																			
**V**	0.64**	0.93**																		
**AIL**	0.82**	0.29	0.55**																	
**E**	0.63**	0.21	0.39*	0.47**																
**gs**	0.63**	0.18	0.36*	0.46**	0.99**															
**A**	0.47**	0.21	0.32*	0.36*	0.78**	0.81**														
**iWUE**	-0.39*	0.04	-0.12	-0.33**	-0.41*	-0.38*	0.14													
**ADM**	0.79**	0.57**	0.77**	0.55**	0.65**	0.62**	0.48**	-0.25												
**RDM**	0.49**	0.35*	0.46**	0.29	0.28	0.32*	0.31	-0.02	0.56**											
**RL**	0.33*	0.09	0.25	0.22	0.08	0.04	-0.03	-0.02	0.41*	0.25										
**R:S**	-0.56**	-0.31	-0.47**	-0.41**	-0.53**	-0.48**	-0.38*	0.26	-0.69**	0.12	-0.17									
**TDM**	0.78**	0.56**	0.75**	0.52**	0.61**	0.61**	0.47**	-0.21	0.97**	0.76**	0.40*	-0.51**								
**δ^13^C**	0.51**	0.24	0.38*	0.41**	0.21	0.22	0.31	-0.06	0.43**	0.48**	0.03	0.26	0.51**							
**GA3ox**	0.11	0.16	0.16	0.35*	-0.06	-0.04	-0.07	-0.04	0.02	0.03	-0.15	0.06	0.03	0.34*						
**ABA**	0.31	0.03	0.13	0.42**	0.08	0.12	0.12	-0.11	0.08	0.02	-0.07	-0.06	0.07	0.41**	0.63**					
**PA**	0.24	-0.01	0.06	0.34*	0.11	0.12	0.03	-0.21	0.02	-0.04	-0.04	-0.03	0.01	0.21	0.37*	0.76**				
**ZOG**	0.18	-0.11	-0.03	-0.09	0.09	0.12	0.01	-0.17	0.15	0.16	0.01	-0.11	0.17	0.22	0.06	-0.07	-0.14			
**iPR**	0.09	-0.06	-0.01	0.09	-0.11	-0.07	-0.13	0.05	0.13	0.15	0.09	0.01	0.15	0.02	-0.13	-0.05	-0.16	0.11		
**IAA**	0.41*	-0.04	0.11	0.48**	0.14	0.12	0.06	-0.21	0.26	0.06	0.05	-0.24	0.22	0.42**	0.37*	0.42**	0.38*	0.21	0.31	
**GA3**	-0.15	-0.16	-0.14	-0.21	-0.01	0.02	-0.23	-0.24	-0.04	-0.12	-0.12	0.09	-0.07	-0.35*	-0.28	-0.27	-0.02	0.05	0.33*	-0.2

^+^H, Height; D, Diameter; V, Volume; AIL, Apical Internode Length; E, transpiration; gs, stomatal conductance; A, photosynthesis; iWUE, intrinsic water use efficiency; ADM, Aboveground Dry Biomass; RDM, Root Dry Biomass; RL, Root Length; R:S, Root:Shoot ratio; TDM, Total Dry Biomass; GA3ox, Gibberellin 3-oxidase gene expression; ABA, Abscisic acid; PA, Phaseic acid; ZOG, Zeatin-O-glucoside (trans); iPR, Isopentenyladenosine; IAA, Indole-3-acetic acid; GA3, Gibberellin 3. Asterisks and gray shadows indicate significant values at *P ≤ 0.05 and **P ≤ 0.01.

### Interaction effects of breeding strategies and growth groups on growth, biomass, gas exchange, hormones and expression of GA-related genes

**Figure 4:**
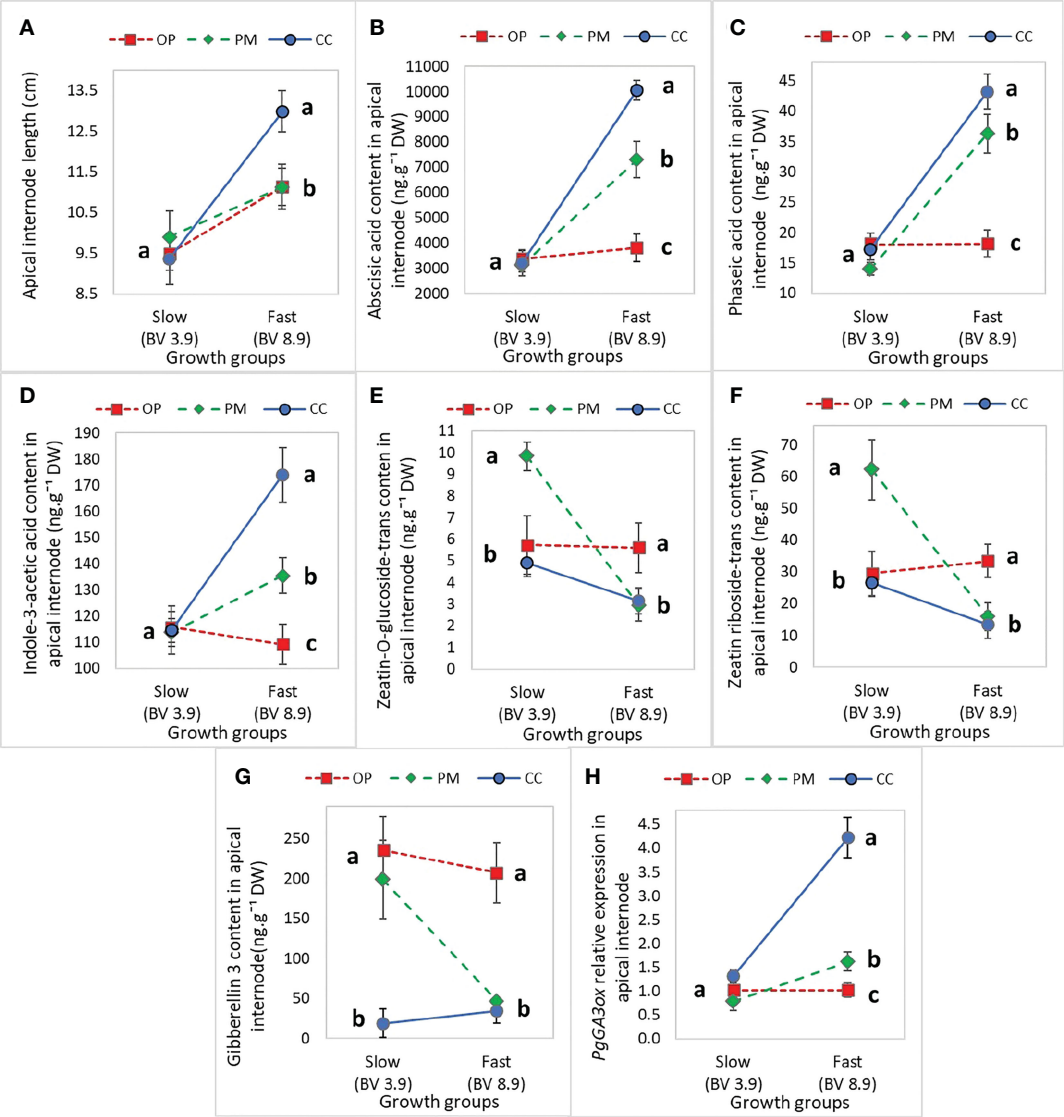
Mean (± SE) effect of the interaction between ‘Growth group’ and ‘Breeding strategy’ on growth and hormone levels in the apical internode in 2-year-old white spruce seedlings for **(A)** The Apical internode length (n=120, 10 blocks, 12 families), **(B)** Abscisic acid glucose ester in the apical internode, **(C)** Phaseic acid in the apical internode, **(D)** Indole-3-acetic acid, **(E)** Zeatin-O-glucoside-trans, **(F)** Zeatin riboside-trans, **(G)** *PgGA3ox* gene expression, and **(H)** GA3 hormone level (n=36, 3 blocks, 12 families). The ‘Growth groups’ are slow (F193+F122) and fast (F927+F138), and the ‘Breeding strategies’ are OP (open pollination), PM (polymix pollination), and CC (controlled crosses) (see Materials and Methods). Mean values are represented by squares (open pollination), triangles (polymix pollination) and circles (controlled crosses). *PgTIF5A* (GenBank accession number DR448953) was used as the control gene. Letters indicate differences (mean) between the breeding strategies using all values from the growth groups (fast, slow) with a Tukey’s test, at 95% confidence level. Last measurements and harvesting were done on Day 92 (28 April 2020).

Height, diameter, volume, and root length did not show statistically significant main or interaction effects, but apical internode length showed a main effect for growth group (P<0.01) and an interaction effect (P=0.03) (**Table 5**). Also, above ground dry biomass, root:shoot ratio, iWUE and δ^13^C showed statistically significant main effects for growth groups and breeding strategies with no interaction effects (**Table 5**). Regarding hormone levels and gene expression, abscisic acid glucose ester, phaseic acid, zeatin-O-glucoside-trans, zeatin riboside-trans, indole-3-acetic acid, gibberellin 3, and *PgGA3ox1* showed statistically significant main effects for breeding strategy and interaction effects (**Table 6**). Controlled crosses from the fast-growth group showed the largest apical internode length, and higher amounts of ABA, PA, IAA, and *PgGA3ox* gene expression, compared to the slow-growth group (**Figures 4A–D, G**). Controlled cross pollination and PM showed lower levels of zeatin in the fast-growth group compared to the slow-growth group (**Figures 4E, F**). In general, open pollination showed similar hormone levels of ABA, PA, IAA, zeatin, GA3, *PgGA3ox* expression for the fast and slow growth groups, but presented a higher apical internode length in the fast-growth group compared to the slow-growth group (**Figure 4**). Also, open pollination showed a statistically significant higher root:shoot ratio, iWUE and δ^13^C compared to polymix pollination and controlled crosses (**Supplementary Material 7**).

**Table 5:** Results (*P*-values) from the ANOVA mixed model analysis of ‘Growth group’ fixed-effect (fast and slow growth), ‘Breeding strategy’ fixed-effect (open pollination, polymix pollination, controlled crosses), and ‘Growth group’ by ‘Breeding strategy’ interactions on growth, biomass and gas exchange traits (n=120, 10 blocks, 12 families) in 2-year-old white spruce seedlings.

Traits	Growth group	Breeding strategy	Growth group x Breeding strategy
Height	0.109	0.349	0.881
Diameter	0.626	0.583	0.995
Volume	0.396	0.297	0.887
Apical internode length	<0.01**	0.164	0.032*
Root length	0.606	0.113	0.388
Above ground dry biomass	0.041*	0.183	0.782
Root dry biomass	0.714	0.952	0.758
Root-Shoot ratio	0.013*	0.031*	0.298
Total dry biomass	0.232	0.339	0.958
Photosynthesis	0.399	0.921	0.552
Transpiration rate	0.361	0.217	0.916
Stomatal conductance	0.366	0.243	0.962
Intrinsic water use efficiency	0.686	0.034*	0.181
δ^13^C	0.181	0.038*	0.824

Families 927 and 138 were used for the ‘fast’ category, and families 193 and 122 were used for the ‘slow’ category, each with 30 trees. Asterisks and shaded cells indicate significant values at *P ≤ 0.05 and **P ≤ 0.01. Last measurements and harvesting were done on Day 92 (28 April 2020).

**Table 6:** Results (*P*-values) from the ANOVA mixed model analysis of ‘Growth group’ fixed-effect (fast and slow growth), ‘Breeding strategy’ fixed-effect (open pollination, polymix pollination, controlled crosses), and ‘Growth group’ by ‘Breeding strategy’ interactions on hormone levels and expression of GA genes (n=36, 3 blocks, 12 families) in 2-year-old white spruce seedlings.

Traits	Growth group	Breeding strategy	Growth group x Breeding strategy
Abscisic acid (ABA)	0.013*	0.518	0.647
Abscisic acid glucose ester (ABA-GE)	<0.01**	0.041*	0.012*
Phaseic acid (PA)	0.015*	0.035*	0.043*
7’-hydroxy-abscisic acid (7’OH-ABA)	0.825	0.631	0.287
Zeatin-O-glucoside-trans (ZOG-t)	<0.01**	0.024*	<0.01**
Zeatin-O-glucoside-cis (ZOG-c)	0.014*	0.829	0.426
Zeatin riboside-trans (ZR-t)	0.034*	0.037*	0.039*
Dihydrozeatin riboside (dhZR)	0.061	0.364	0.366
Isopentenyladenosine (iPR)	<0.01**	0.031*	0.238
Indole-3-acetic acid (IAA)	0.011*	<0.01**	0.032*
Gibberellin 3 (GA3)	0.061	0.039*	0.039*
*PgGA3ox1*	0.033*	0.024*	0.027*
*PgGA20ox1*	0.508	0.741	0.655
*PgDELLA1*	0.701	0.502	0.387
*PgGID1*	0.781	0.626	0.464

The levels of the 11 hormones and gene expression of the four genes were measured in the apical internode (see Materials and Methods). Families 927 and 138 were used for the ‘fast’ category, and families 193 and 122 were used for the ‘slow’ category, each with 30 trees. Asterisks and gray shadow indicate significant values at *P ≤ 0.05 and **P ≤ 0.01. Last measurements and harvesting were done on Day 92 (28 April 2020).

### Gene expression of GA-related genes in the apical internode of white spruce seedlings from three different breeding strategies

**Figure 5:**
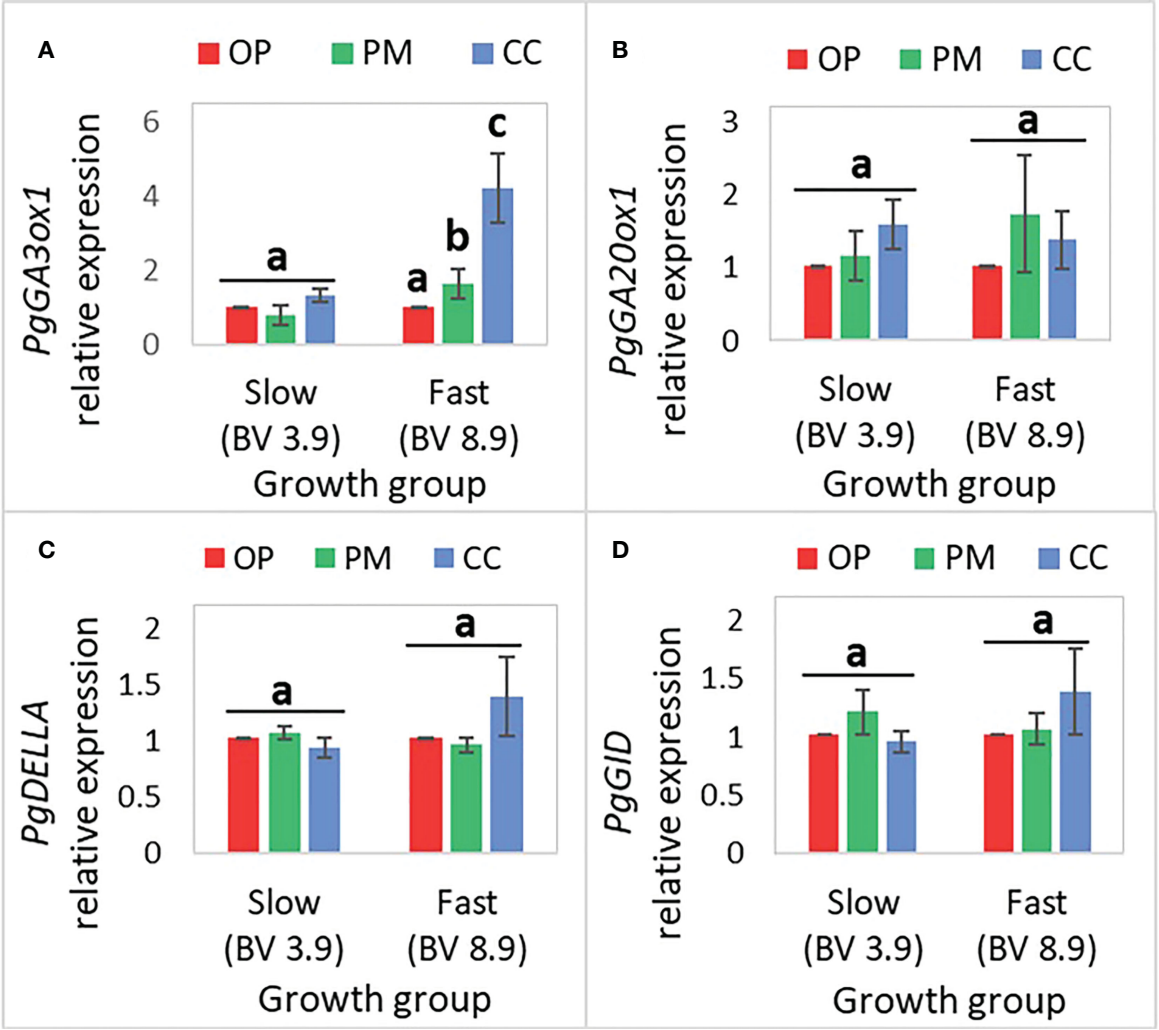
Mean (± SE) of the relative expression of GA-related genes in the apical internode (n=36, 3 blocks, 12 families) in 2-year-old white spruce seedlings for **(A)** *PgGA3ox1*, **(B)** *PgGA20ox1*, **(C)** *PgDELLA*, **(D)** *PgGID*. The ‘Growth groups’ are slow (F193+F122) and fast (F927+F138), and the ‘Breeding strategies’ are OP (open pollination), PM (polymix pollination), and CC (controlled crosses) (see Materials and Methods). *PgTIF5A* (GenBank accession number DR448953) was used as the control gene. Letters above bars indicate differences (mean) between the breeding strategies in each of the growth groups (fast, slow) with a Tukey’s test, at 95% confidence level. Last measurements and harvesting were done on Day 92 (28 April 2020).

The *PgGA20ox*, *PgDELLA*, and *PgGID* gene expression did not show statistically significant differences between OP, PM and CC, in the fast- and slow-growth groups (**Figure 5**; **Table 6**). The only statistically significant difference was found for *PgGA3ox1* gene expression between breeding strategies, for the fast-growth group, with CC exhibiting 4-fold more expression than OP (**Figure 5A**). Also, the *PgDELLA* and *PgGID* gene expression was 1.5-fold higher in CC than OP and PM for the fast-growth group, which was not statistically significant (**Figures 5C, D**).

### Principal components analysis for growth and molecular traits among the breeding strategies and growth groups

**Figure 6:**
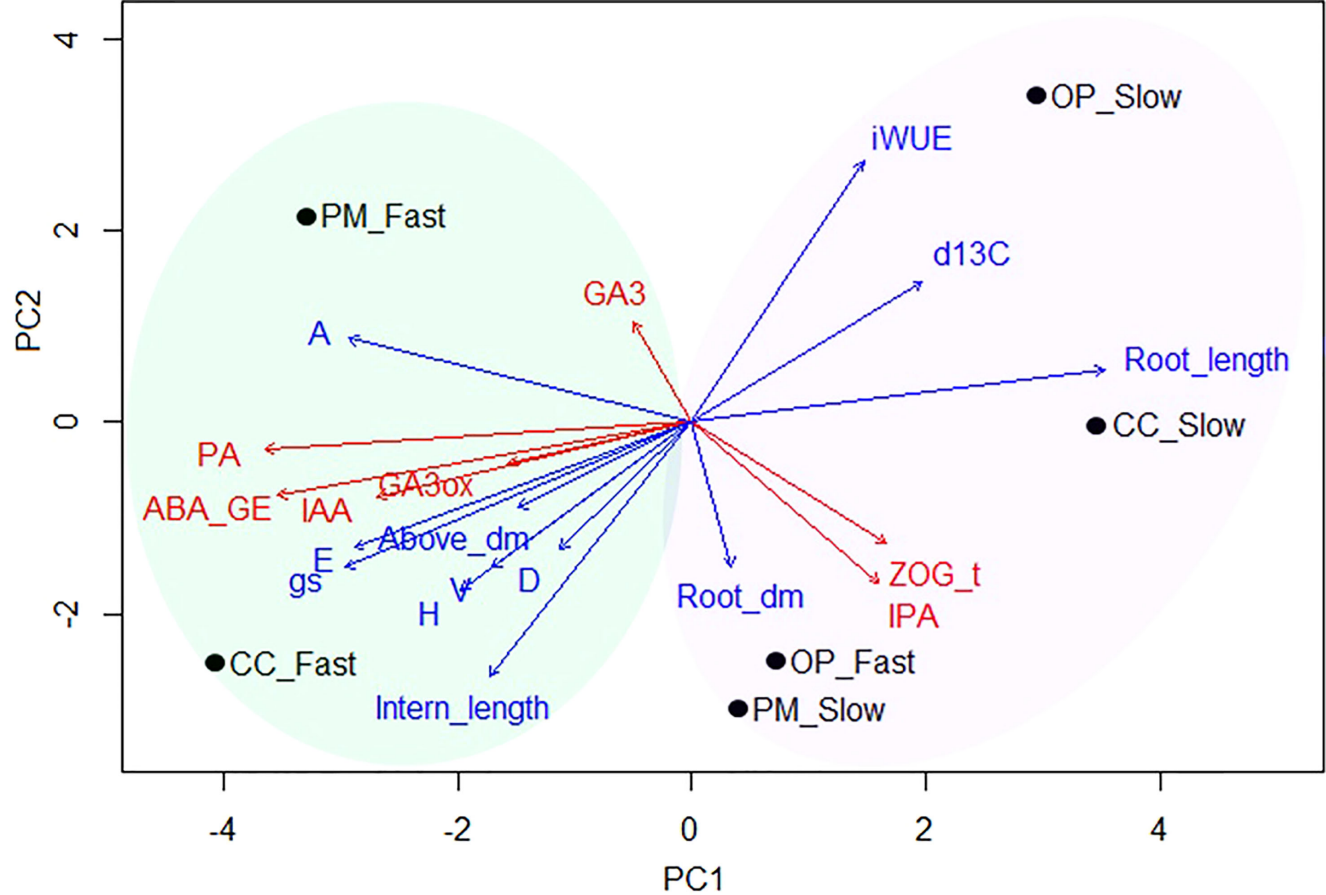
Principal component analysis (PCA) biplot of combined data sets with post-hoc fit of growth and gas exchange traits (PC1 = 44.2%, blue arrows), and molecular traits (PC2 = 29.4%, red arrows) over the six groups (black dots) (n=36, 3 blocks, 12 families). The vector lengths represent the importance of each variable along the two plotted axes in relation to all possible principal components, while distances between vectors and families indicate how much variation is explained by each vector. Blue arrows: H, height; D, diameter; V, volume; Intern_length, apical internode length; A, photosynthesis; E, transpiration; gs, stomatal conductance; iWUE, intrinsic water use efficiency; Above_dm, above ground biomass; Root_dm, root biomass; Root_length, root length; d13C, δ^13^C isotopes. Red arrows: PA, Phaseic acid; ABA_GE, Abscisic acid glucose ester; IAA, Indole-3-acetic acid; ZOG_t, Zeatin-O-glucoside-trans; IPA, Isopentenyladenosine; GA3, Gibberellin 3; GA3ox, *PgGA3ox1* gene expression. Groups: CC_Fast, controlled crosses with fast growth (F927+F138); CC_Slow, controlled crosses with slow growth (F193; F122); PM_Fast, polymix pollination with fast growth (F927+F138); PM_Slow, polymix pollination with slow growth (F193; F122); OP_Fast, open pollination with fast growth (F927+F138); OP_Slow, open pollination with slow growth (F193; F122).

For this analysis, we considered six groups for clustering: OP, PM and CC pollination in the fast- and slow-growth groups (**Figure 6**). The first principal component associated with growth, biomass and gas exchange parameters explained 44.2% of the variance, whereas the second principal component associated with the molecular traits explained 29.4% of the variance (**Figure 6**). The position of the six groups in the PCA quadrant shows two clear patterns in the growth, physiological and molecular mechanisms (**Figure 6**). The first cluster represents the fast-growth for CC and PM, with the highest values for height, diameter, volume, apical internode length, above ground dry biomass, A, E, gs, PA, ABA, IAA, GA3, and *GA3ox1* (green oval, **Figure 6**). The second cluster represents all the slow-growth (for OP, CC, and PM) and fast-growth families for OP with the highest values of iWUE, δ^13^C, root length, root dry biomass, zeatin and iPR (purple oval, **Figure 6**). The OP and CC with slow-growth exhibited the lowest productivity above ground with the highest iWUE and root development, while CC with the fast-growth group showed the best productivity with the lowest iWUE and root length (**Figure 6**).

## Discussion

### Genetic variation of trees from different breeding strategies leads to opportunities in selecting new material

The results presented in this study indicate that height, above ground dry biomass and root:shoot ratio are under moderate genetic control (heritabilities between 14-21%) for development of 2-year-old white spruce seedlings from three different breeding strategies (**Table 3**). Furthermore, for total dry biomass, volume and root length, the heritability values were close to 10%, meaning nearly 90% of the variation may be caused by environmental factors, as previously shown (Raj et al., 2006). In trees, narrow-sense heritability for height can range between 0.1 and 0.5 in seedlings at an early stage (Scotti-Saintagne et al., 2004; Sotelo Montes et al., 2007; Tripiana et al., 2007; Bouffier et al., 2008; Callister and Collins, 2008; Ward et al., 2008; Scotti et al., 2010). Heritability of 3-year-old white spruce families from an improvement program in Quebec, Canada ranged between 0.17 to 0.45 for height (Li et al., 1993) and in another Quebec study, heritability was 0.26 for height and 0.14 for diameter (Wahid et al., 2013). In *Pinus rigida*, seedlings at a very young age obtained from trees growing in the Waiden Woods, Massachusetts, showed extensive variation for growth, in contrast to their hypothesis (Raj et al., 2006). Our study also showed heritabilities close to 0 for all gas exchange parameters (**Table 3**), similar to other studies (Scotti-Saintagne et al., 2004; Brendel et al., 2008; Scotti et al., 2010). Divergence in heritability estimates could be partly explained by differences in seedling age and number of families under investigation, and can only be applied to a particular population growing in a particular environment at a particular point in time (Rweyongeza et al., 2010; Carles et al., 2012). The ABLUP values obtained for growth and physiology traits of the white spruce seedlings in our study showed substantial genetic variability between families from the different breeding strategies (**Figure 1**). Our experiment found specific faster- and slower-growing families from OP and CC, with different developmental and physiological characteristics. Previous authors have discussed the possible evolutionary genetic meaning of the natural variation when domesticating conifers (Scotti et al., 2010; Gepts, 2014). Although family 754 showed the highest ABLUP for root:shoot ratio from OP among all families (**Figure 1H**), significant variability in dry root biomass between families and breeding methods is clear (**Figures 1F, H**), showing large genetic variation in radicular biomass allocation and potential nutrient uptake among families, as previously described (Theodorou and Bowen, 1993). The vast genetic variability among all the seedlings analyzed within families and breeding strategies in our work (**Figure 2**) is consistent with previous studies. In trials performed in Quebec, Canada, heights for 2-year-old white spruce clones and seedlings varied from 14.4 to 31.8 cm and from 15.8 to 24.3 cm, respectively (Lamhamedi et al., 2000). In this study, the three seedlings from F138 (polymix pollination) showed a range of 11-16 g for total dry biomass, and the three seedlings from F138 (open pollination) exhibited a range of 32-40 cm for height (**Figure 2**). Large ranges were also found among seedlings from CC and OP in F927 for gas exchange traits, hormone levels and *PgGA3ox1* gene expression (**Figures 2**, **3**). Significant clonal variation was also observed when studying differences of plants from seedlings and clones: for many variables (height, dry biomass of new roots, needle dry biomass and branch density), and differences among clones were significantly greater than differences among seedlings within a family (Lamhamedi et al., 2000). Variation in growth and physiology reflected genetically determined differences among individuals within a family (Lamhamedi et al., 2000). Tree breeding can result in a bigger pool of phenotypic variation that can be managed wisely if the variation is mainly due to genetic rather than environmental effects (Gepts, 2014). These findings suggest there is minimal risk applying any particular breeding strategy in white spruce, as our ability to in fact manipulate the genetics in the first generation is minimal due to the enormous natural variability in these trees. Perhaps our concern with reducing genetic variability, at the expense of gain, is overstated in the early generation breeding stages in conifers, and several generations will be needed, unless a trait is highly heritable (e.g. stem straightness) or has limited variability.

### Trade-offs between growth, carbon allocation and gas exchange of plants obtained from different breeding strategies

Correlation analyses showed a general structure with taller white spruce stems associate with larger biomass, photosynthesis, transpiration, water use efficiency (δ^13^C) and higher levels of hormones (**Table 4**). This seems to hold for both phenotypic and genetic correlations, thus suggesting that these traits share a genetic bases, as previously found in *Sextonia rubra* (Scotti et al., 2010). In general, we found a trade-off in developmental traits between breeding strategies, with controlled crosses from the fast-growth group showing the best apical growth, but in some cases, open pollination from the different growth groups showing the best root development (**Figures 2**–**4**). In our study, some individuals showed a preference to allocate photoassimilates to roots rather than shoots (e.g. three plants from F122, PM and OP, slow growth), but other seedlings showed the best height with a lower root:shoot ratio (e.g. plants from F927 and F138, PM, fast-growth group), which are not suitable for selection and could be at risk of not developing properly when older (**Figure 2C**). On the other hand, some individuals showed greater height, root:shoot ratios close to 1.0, greater stomatal opening, and higher water use efficiency (e.g. one plant from F927 and another from F138, CC, fast growth) (**Figures 2**, **4**, **5**); identifying themselves as the most suitable for selection suggesting both growth and drought resistance could be selected for simultaneously. Differences in developmental and growth partitioning have been reported previously among clones and families of white spruce during initiation, maturation and germination (Park et al., 1993; Park et al., 1994; Lamhamedi et al., 2000). Previous studies indicated that white spruce families exhibiting inferior height growth showed higher net photosynthesis under drought stress than families exhibiting intermediate and superior height growth (Bigras, 2005). Gas exchange and growth usually show a positive correlation at the intraspecific level, but trade-offs between light and carbon acquisition for individuals within a family have also been reported (Scotti et al., 2010). In our study, negative correlations between photosynthesis and height were shown suggesting a tradeoff in allocation of resources (**Figure 2B**). Trees that are genetically predisposed to slower growth, may require less water to maintain adequate evapotranspiration, and could therefore be more tolerant to drought events (Moran et al., 2017; Six et al., 2021). As in all tree species, the rate of photosynthesis and stomatal conductance in spruce trees is influenced by light, temperature, atmospheric humidity, CO_2_ concentration, soil water availability and phenology (Lamhamedi and Bernier, 1994; Hébert et al., 2011). In our study, white spruce seedlings showed stomatal conductance values from 0.065 to 0.077 mol H_2_0 m^-2^ s^-1^, higher than that reported in 2-year-old black spruce seedlings (0.03 mol H_2_0 m^-2^ s^-1^) grown in outside sand beds (Lamhamedi and Bernier, 1994). Stomatal conductance influences net photosynthesis by controlling the amount of CO_2_ that can enter the mesophyll, with stomatal limitation to net photosynthesis becoming important only at low values of stomatal conductance (Lamhamedi and Bernier, 1994; Hébert et al., 2011). Furthermore, root growth in spruce trees has been shown to decline during the period of shoot growth, as shoot growth itself uses most of the stored and current photosynthates, but at other times of the year, soil temperature is the major regulator of root growth (Lamhamedi and Bernier, 1994). Trees with higher root development generally show higher photosynthesis rates and such individuals can re-establish soil–root contact more rapidly than poor-rooting trees following transplanting (Lamhamedi et al., 2000). It has been described that *Picea* species overcome root deformation by forming adventitious roots after planting, which are critical for water and nutrient uptake, whereas deeper roots are more essential for stability and coping during drought episodes (Lamhamedi et al., 2000).

### Trade-offs between hormones levels and gene expression of plants obtained from different breeding strategies

This study showed that *PgGA3ox1* expression was higher in fast vs. slow groups, as reported in previous experiments (Galeano and Thomas, 2020). *PgGA3ox1* showed the highest expression among the other GA-related genes (i.e. *PgGA20ox1*, *PgDELLA*, *PgGID*) as observed in one of our previous studies (Galeano and Thomas, 2020). *PgGA3ox1* is, again, showing a strong influence in growth compared to other GA-related genes, particularly in fast-growing individuals. In general, our study found strong correlations between apical internode length, ABA, PA levels (one of ABA’s catabolites), IAA, *PgGA3ox* gene expression, and significant correlations of GA_3_ with δ^13^C and iPR (**Table 4**). In particular, we found a pattern of greater accumulation of *PgGA3ox*, IAA, ABA, PA in controlled crosses in the fast-growth group compared to the other breeding strategies (**Figures 3**, **4**, **6**). In addition, longer root length, root dry biomass, ZOG_t, and iPR levels were higher in OP material, and the slow-growth group (**Figure 6**). Our study suggests a combined effect of PA (terpenoid), IAA (auxin), ABA, GA_3_ in stem elongation of white spruce seedlings, and high amounts of IAA could potentially be inhibiting Zeatin synthesis, as previously described (Lulsdorf et al., 2013; Dilworth et al., 2017; Lorrai et al., 2018; Shu et al., 2018; Akhtar et al., 2020). Previous studies in white spruce seedlings also showed different profiles for ABA, cytokinins, auxin and expression of GA-related genes related to bud development (Kayal et al., 2011). In this study, GA_3_ hormone content could potentially experience feedback regulation of *PgGA3*, and be regulated by ABA in improved material (e.g. controlled crosses), since ABA and GA antagonistically regulate stem elongation (Cline and Harrington, 2007; Lorrai et al., 2018; Shu et al., 2018; Akhtar et al., 2020). In unimproved material (open pollination), it is possible that regulation is not as strong as in improved seedlings. In Arabidopsis, a disruption of four genes encoding iPR by T-DNA insertion showed failure to form cambium and reduced thickening of the root and stem, demonstrating the relevance of cytokinins for normal development of both the root and shoot (Matsumoto-Kitano et al., 2008). Previous studies have explained that family differences in expression of gibberellin-related genes and endogenous hormone levels may explain much of the natural variation in tree stem growth capacity (Stoehr et al., 1998; Park et al., 2014). *PgGA3ox*, IAA, ABA, PA profiling combined with the measurement of growth and physiological traits could have the potential to accelerate the selection of spruce families at an early stage for rapid growth, as previously suggested (Park et al., 2014; Galeano and Thomas, 2020). Consequently, the identification and monitoring of hormone levels and expression of GA-related genes that control traits relevant to tree domestication is a powerful tool, especially as our knowledge of tree-specific processes is still insufficient and in its infancy (Singh et al., 2021). In any event, other plant hormones not studied here could show correlations between their concentration levels, gene expression, and growth parameters in spruce seedlings.

### Implications of this study

The term “Physiological breeding strategy” was recently introduced, which was successfully adapted in legume crop improvement to narrow the gap between breeders and physiologists through collaborative approaches to understand complex traits, potential of physiology based approaches and how they impact yield gains and abiotic stress tolerance in these crops (Shunmugam et al., 2018). Certainly, tree breeders and physiologists have the challenge of studying and understanding the phenotypic plasticity of simple and complex developmental traits, the relationship among them, and their importance as determinants of growth of mature forest trees (Raj et al., 2006; Singh et al., 2021). Therefore, early selection of superior clones based on both physiological and morphological characteristics is feasible (Stoehr et al., 1998; Lamhamedi et al., 2000). In Quebec, Canada, the identification of strong positive genetic correlations allowed diameter to be used as an effective method for indirect selection of white spruce seedlings with heavier root systems (Carles et al., 2012). In Ontario, Canada, root establishment and survival in 2-year-old *Pinus resinosa* seedlings were positively correlated with the length of needles (Paterson and Faylel, 1984). In Georgia, USA, loblolly pine seedlings from a fast-growing provenance showed higher rates of net photosynthesis than seedlings from slow-growing provenances indicating the potential of this physiological trait for selection (Boltz et al., 1986). In our study, several seedlings had both greater root growth and high stem elongation rates (**Figure 1**), indicating that selection of white spruce trees based on root growth would not exclude genotypes with greater above ground growth, as described in previous studies (Lamhamedi et al., 2000). “Tree diversity breeding” is another new approach that seeks to amplify biodiversity in production systems to address global challenges and maximize linkages between existing tree breeding methods (Graudal et al., 2022). Therefore, significant investments in tree breeding offers the prospect of good economic returns, frequently greater than those from alternative forestry investments, along with good silviculture, management and product processing (Kanowski and Borralho, 2004). Future research should evaluate the response of trees, produced from different breeding strategies, to stressful conditions. It is also very likely that studies in biotechnology, phenomics, genomics, genome-wide associations and epigenetics will become essential methods in tree improvement programs, forest management and conservation practices since they allow for the identification of causal variants underlying phenotypes of interest, the evolutionary trajectory of populations to be studied, and help us understand the phenotypic plasticity and adaptive capacity of trees (Faino and Thomma, 2014; Plomion et al., 2016; Lind et al., 2018; Sow et al., 2018; Ebrahimi et al., 2020; Mahony et al., 2020; Rellstab, 2021; Singh et al., 2021). Compared with agricultural crops, timely domestication of trees is almost unachievable through traditional genetic improvement methods alone, due to the long breeding cycles and rotation times (Pearsall, 2008; Harfouche et al., 2012).

## Conclusions

The genetic architecture of parameters such as biomass and gas exchange in white spruce have not been widely studied, particularly in families obtained from controlled crosses. We determined that height, above ground dry biomass, root length and root:shoot ratio traits are heritable, whereas diameter and all gas exchange traits studied appear to be under considerably less genetic control in the 2-year-old white spruce seedlings we studied. Based on the ABLUP values, certain families performed consistently better for both growth and physiology traits generated from polymix pollination and controlled crosses, however, the specific performance of individual families was unpredictable. Interestingly, within each genotype, some growth and physiological traits were similar across breeding strategies, indicating strong genetic control. We found substantial genetic variability in growth, gas exchange, hormone levels and gene expression traits between seedlings within families and breeding strategies when plotting the individual values. Furthermore, we observed trade-offs between growth, carbon allocation, photosynthesis, hormone levels and gene expression within seedlings. Some individuals showed greater height, more biomass in roots than shoots, higher stomatal conductance (stomata were more open), and higher water use efficiency, a series of traits desirable for selection. Controlled crosses from the fast growth group showed the best apical growth, but in some cases, open pollination from the fast and slow growth groups showed the best root development and higher water use efficiency, based on iWUE and δ^13^C. We also found a pattern of greater accumulation of *PgGA3ox*, IAA, ABA, and PA in controlled cross seedlings with greater growth compared to seedlings from the other breeding strategies and found higher root length, root dry biomass, ZOG_t level, iPR level in open-pollinated seedlings with slower growth. ABA, PA, IAA and *PgGA3ox* actively regulated and promoted apical growth in the controlled cross seedlings and their levels potentially inhibited Zeatin synthesis. Cytokinins could be synergistically working with GA_3_ balancing the root:shoot ratios, while promoting more root development in open-pollinated trees. In this study, we confirmed that hormone levels and expression of GA-related genes have a strong influence on many traits and differences were found between families coming from different breeding strategies. Our findings also show the existence of specific faster- and slower-growing families from open pollination, polymix pollination and controlled-crosses, with different developmental and physiological characteristics. Advancing tree domestication through the application of more advanced breeding approaches is supported by our results, encouraging the use of this phenotypic variation to advance tree improvement programs. For management purposes, it appears that selection based on controlled-crossed families would be reasonably efficient for most growth traits, which showed relatively high ABLUP values and moderate heritability values, however, some individuals from open-pollinated families in this first-generation cycle, also showed outstanding performance, particularly for root development. These OP derived individuals should be selected and used for future breeding as we continue to gain information on parental material through both phenotypic, genetic and physiological study.

## Data availability statement

The datasets presented in this study can be found in online repositories. The names of the repository/repositories and accession number(s) can be found in the article/**Supplementary Material**.

## Author contributions

EG and BT contributed to the conception and design of the study. EG and BT organized the database. EG performed the statistical analysis. EG wrote the first draft of the manuscript. EG and BT wrote sections of the manuscript. All authors contributed to manuscript revision, read, and approved the submitted version. All authors contributed to the article and approved the submitted version.
