# Supplementary material for: Unraveling genetic variation among white spruce families generated through different breeding strategies: Heritability, growth, physiology, hormones and gene expression
Source: Front Plant Sci. 2023 Apr 3;14:1052425. doi: 10.3389/fpls.2023.1052425 (PMC10106773; doi:10.3389/fpls.2023.1052425)
Supplement: Supplementary file 1 [file DataSheet_1.docx]

Supplementary Material 1. Selection of the 15 white spruce genotypes from the Region G1 based on the breeding values (% height) representing the (A) low BV (84 genotypes), (B) mid BV (36 genotypes) and high BV (17 genotypes). All 84 genotypes with low BV were removed (rogued) from the G1 clonal seed orchard during Spring 2018 prior to pollen release. With the remaining genotypes, polymix pollination and controlled crosses were performed during Summer 2018: six genotypes were selected as females (193, 122, 754, 129, 927, 128), nine genotypes were selected as males for polymix (199, 756, 991, 752, 115, 1045, 1002, 1047, 966), and six genotypes (asterisks) were selected as males for controlled crosses (756, 991, 752, 1045, 1047, 966) (see Table 1). Also, genotypes 193 and 122 were used as “Slow growth”, and genotypes 927 and 138 were used as “Fast growth”.


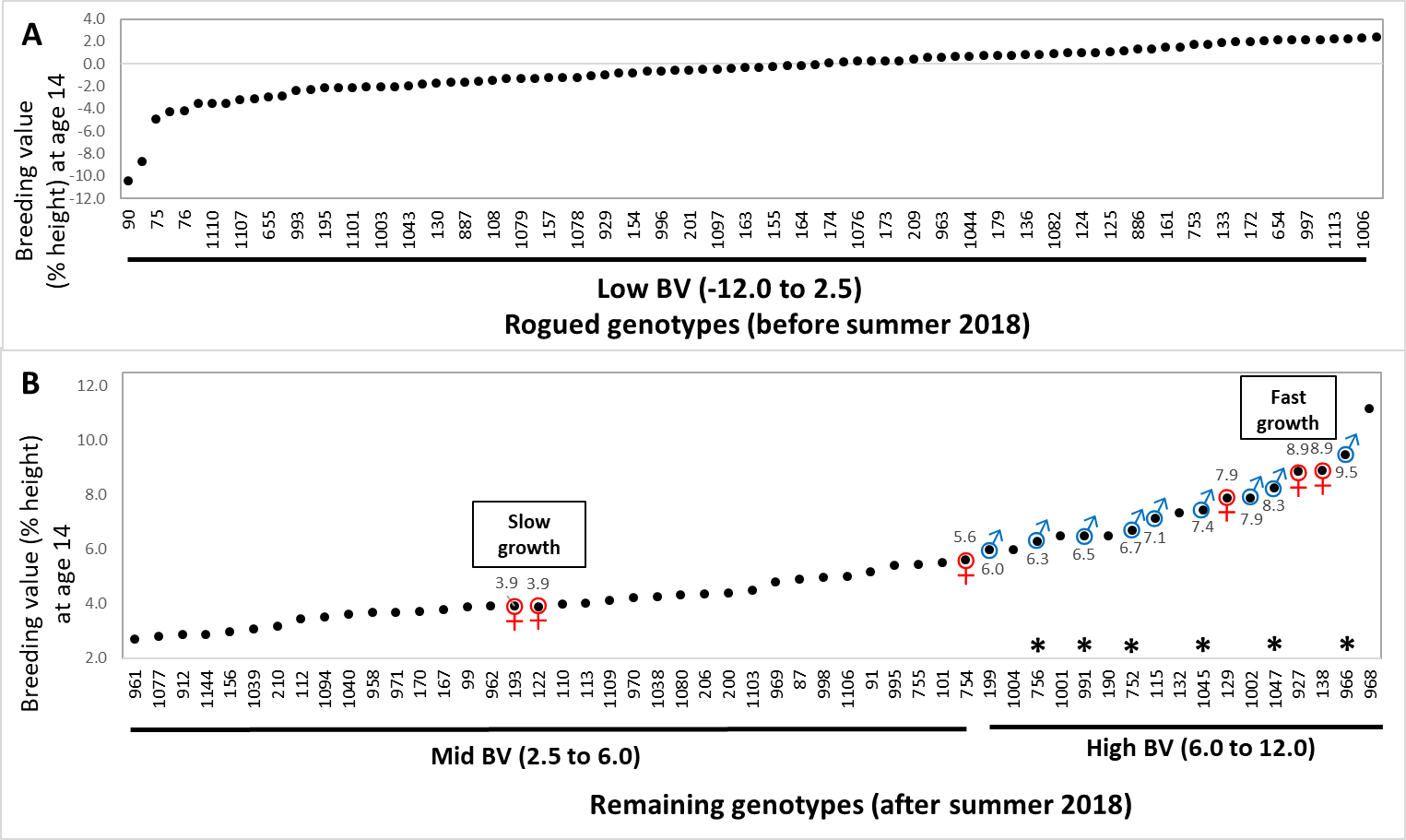


Supplementary material 2. Pictorial representation of conducting controlled crosses in the white spruce G1 clonal seed orchard, Alberta, Canada. a) Initial examination of female strobili for breeding (1st week of May 2018); b) bagging female strobili (2nd week of May 2018); c) male strobili ready for pollen collection (3rd week of May 2018); d) grinding male strobili using funnels and sieves (3rd week of May 2018); e) pollination using a long nozzled squeeze bottle and needle (3rd week of May 2018); f) removing the pollination bags (mid-June 2018), g) cones one month after pollination (mid-July 2018); h) i) j) cone inspection (late June to July 2018); k) cone collection using buckets (mid-August 2018); l) cone packing using small burlap bags (mid-August 2018); m) cones drying and opening (late August 2018); n) tapping cones to remove seeds (end of August 2018); o) seeds attached to wings; p) squashing material by hand to release seeds from wings; q) sieving seeds to remove debris and husks (wings); r) blowing seeds to remove aborted/unfertilized seed coats (end of August 2018). In Alberta, dates for spring and summer are considered from March 19 - June 20 and June 21 - September 23, respectively.


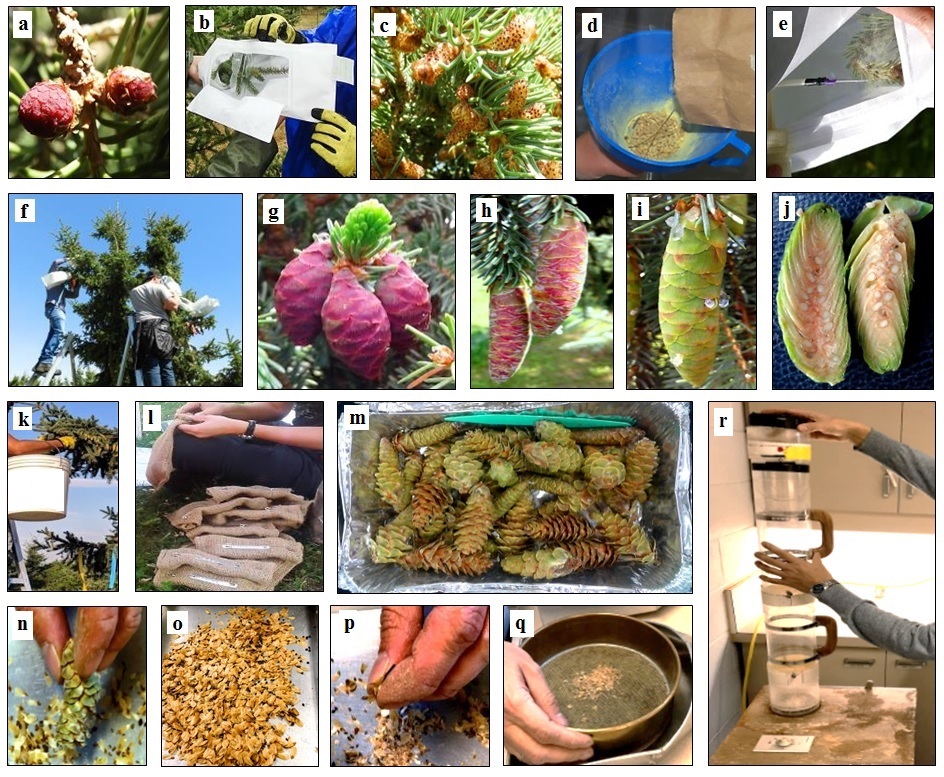


Supplementary material 3. Two-year old white spruce seedlings in the greenhouse at the University of Alberta. Last measurements and harvesting were done at day 92 (April 28, 2020). The experimental design was a randomized complete block design. For the first statistical analysis (BLUPs using the family genetic model), we considered the “BV group” as a factor with two levels (Mid BV and High BV) with 10 blocks, 18 genotypes (n=180). For the second statistical analysis (fixed-effect mixed model), we considered the “growth group” as a factor with two levels (slow growth and fast growth) with 3 blocks, 12 genotypes (n=120). Genotypes were randomly placed in each block. The distance between plants was 10 cm, and each plant was growing in black plastic nursery containers of 2 litres.


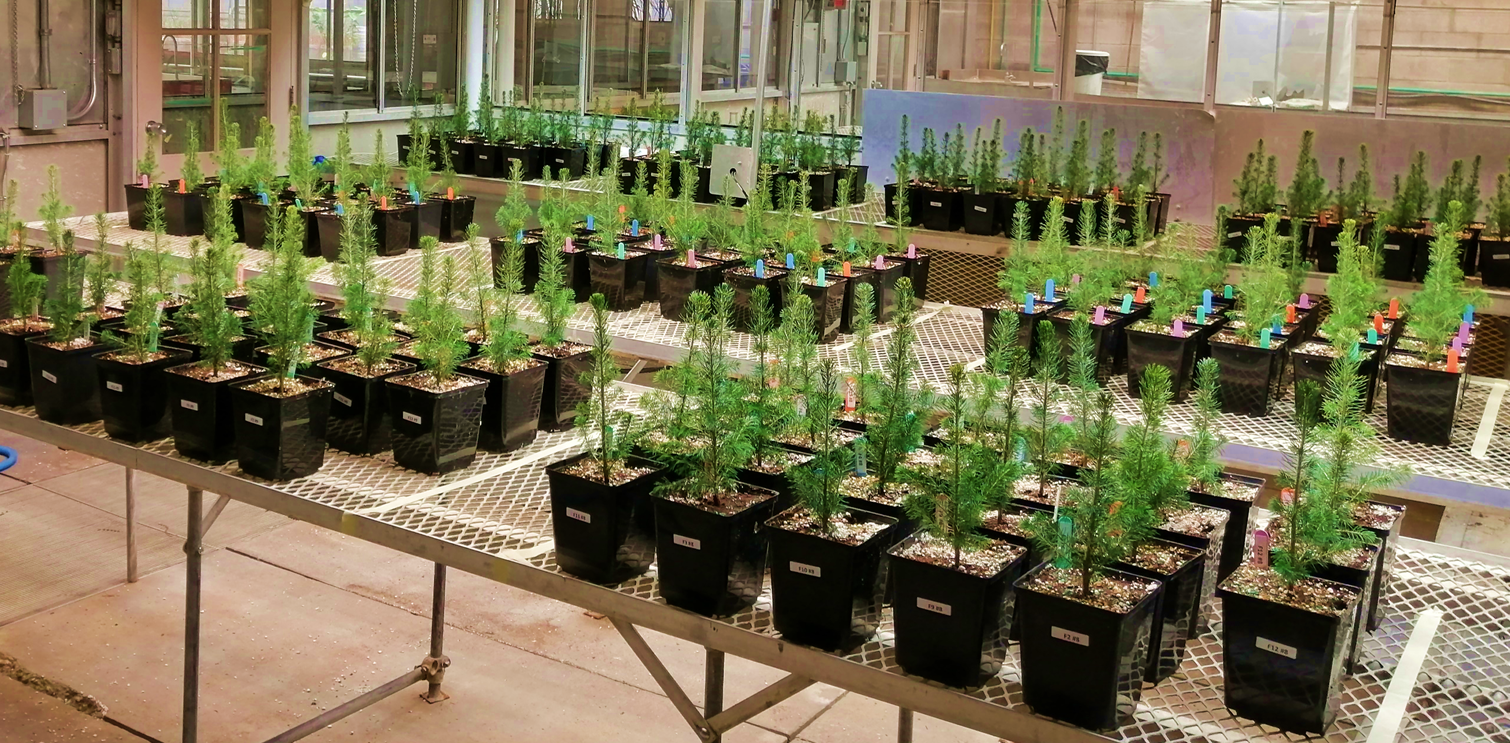


Supplementary material 4. Harvesting of apical internode from 36 trees. A) Use of the scalpel for removing needles from the apical internode in white spruce seedlings; B) Visualization of the apical internode before being harvested; C) Apical internode from two repetitions (#4 and #7), genotype F11, that were placed in different aluminum foils; D) Each apical internode were chopped into 8-10 pieces in order to facilitate the grinding process afterwards; E) Each aluminum foil was labelled inside and outside, F) Each aluminum foil, containing the chopped apical internode, was quickly placed in liquid nitrogen, and then stored at -80°C for further grinding, RNA extraction and hormone analysis.


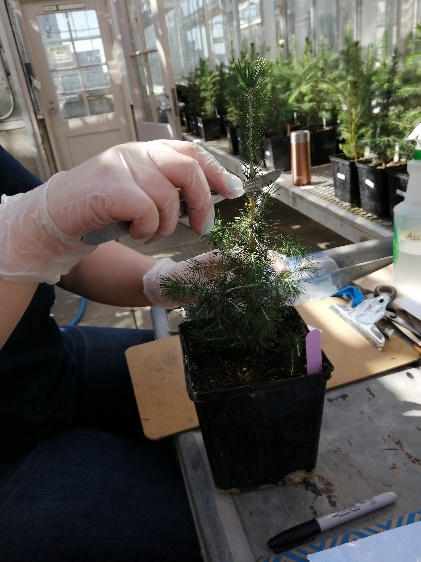

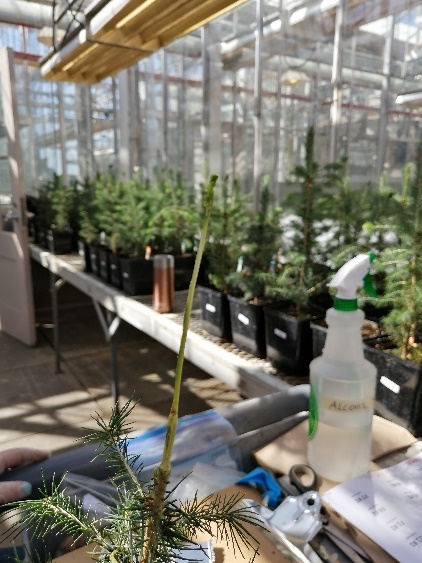

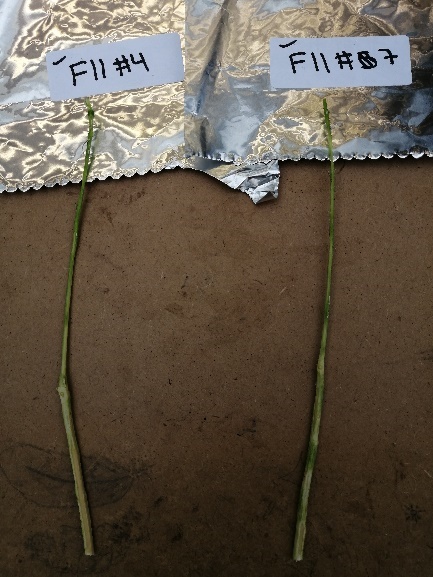

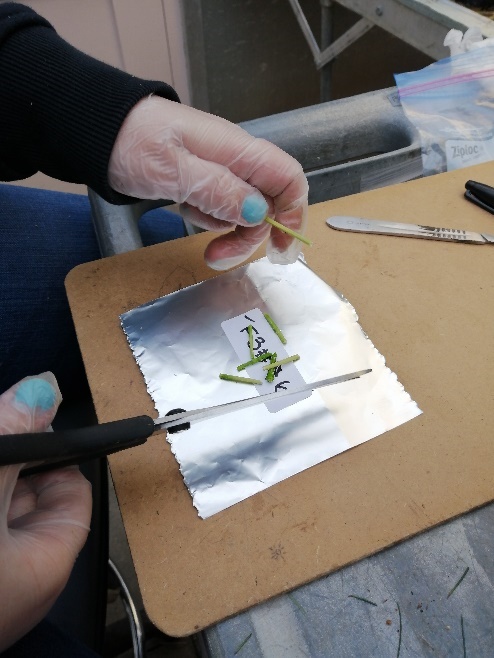

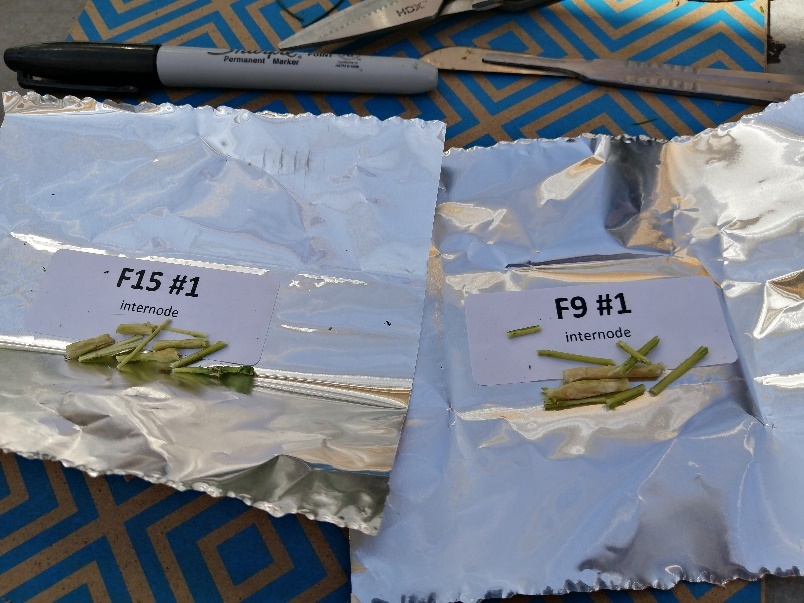

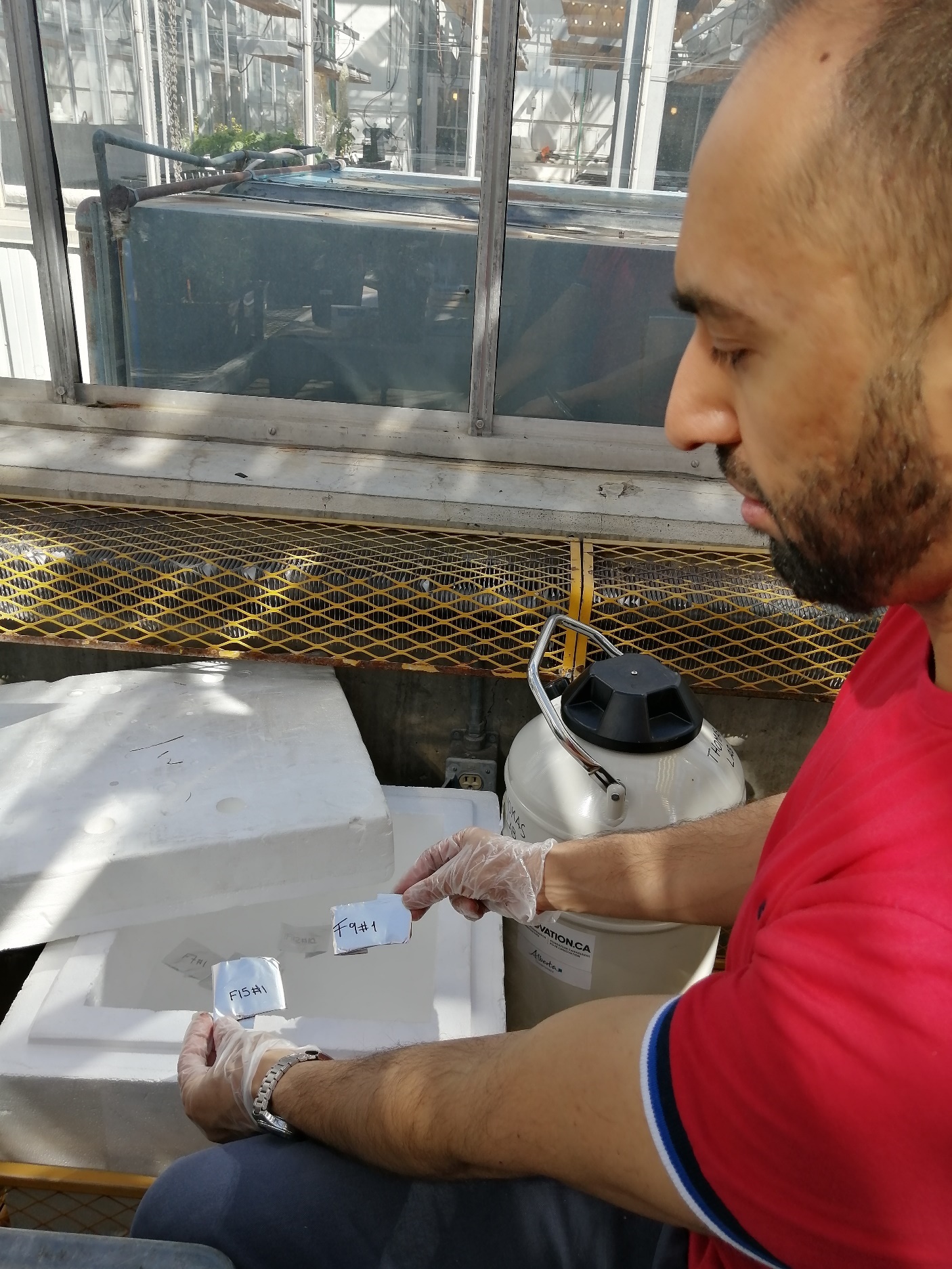


**B**

**C**

**D**

**E**

**F**

**A**

Supplementary material 5. Primers used for the gene expression quantification through real-time PCR. The table shows each gene and primer names, primer sequences, number of nucleotides, temperature of melting, amplicon length and accession number at the NCBI. Genes and primers were selected from previous work (Galeano and Thomas 2020).

| **Gene** | **Primer name** | **Primers for RT-PCR** | **# nt** | **Tm** | **Amplicon**  **length** | **Accession number** |
| --- | --- | --- | --- | --- | --- | --- |
| *PgGA3ox1* | *PgGA3ox1-F* | 5’ CACAGAGCAGTGGCTAACAGA 3’ | 21 | 64 °C | 78 bp | PG29_BFMXY_Trinity_  Pasafly_comp427203_  c0_seq1 |
|  | *PgGA3ox1-R* | 5’ GATAACATCACGAGGTGGAGGC 3’ | 22 |  |  |  |
| *PgGA20ox1* | *PgGA20ox1-F* | 5’ GACACTCGTGAGGTTGACTTTGAT 3’ | 24 | 63 °C | 61 bp | PG29_BFMXY_Trinity_  Pasafly_comp413321_  c0_seq1_rc |
|  | *PgGA20ox1-R* | 5’ CGACGGGTGGCATTGAA 3’ | 17 |  |  |  |
| *PgDELLA1* | *PgDPL1-F* | 5’ CCTAACACTTGGTTGGCACAGC 3’ | 22 | 67 °C | 86 bp | HQ408573.1 |
|  | *PgDPL1-R* | 5’ ACACCTATCACCTTCTTCACGG 3’ | 22 |  |  |  |
| *PgGID1* | *PgGID1-F* | 5’ GCAGGCTCGATGGCAAATA 3’ | 19 | 63 °C | 68 bp | BN001188.1 |
|  | *PgGID1-R* | 5’ CGGCAAGAAGGCATTCCA 3’ | 18 |  |  |  |
| *PgTIF5A1* | *PgTIF5A1.1-F* | 5’ CAAAAAGAAGTGAATGTTCAAGCAA 3’ | 25 | 62 °C | 65 bp | BT102965.1 |
|  | *PgTIF5A1.1-R* | 5’ CACACAAAATGCAGCAAGCA 3’ | 20 |  |  |  |

Supplementary material 6. Melting and standard curves for the five genes used in the study. The temperature of melting for each set of primers (melting curves) and the quantity of transcripts (standard curve) are all included in the graphics.


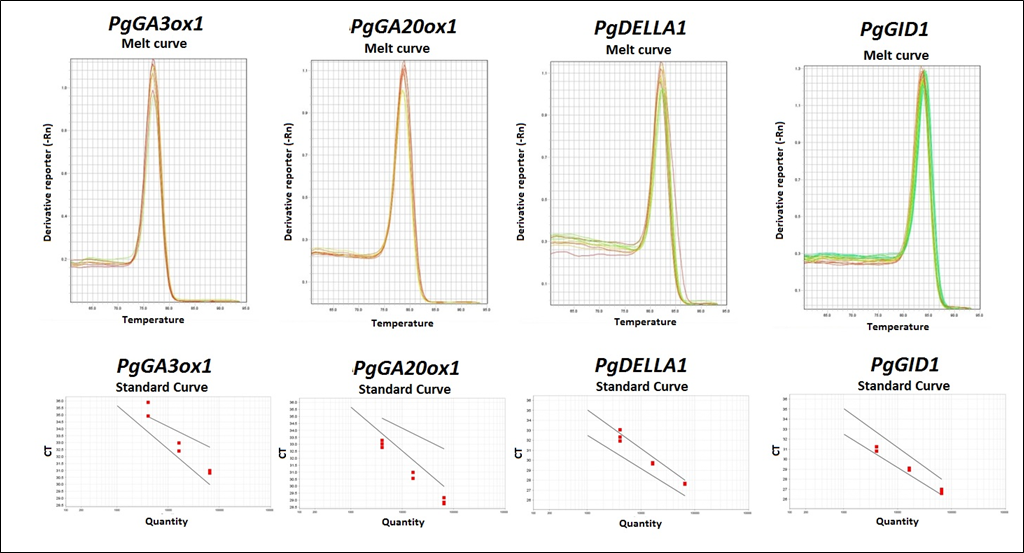


Supplementary Material 7. Main effect (mean ± SE) of the ‘Breeding strategy’ on (A) Root:shoot ratio, (B) iWUE and (C) δ^13^C. The ‘Breeding strategies’ are OP (open pollination), PM (polymix pollination), and CC (controlled crosses). Measurements were taken after 92 days of growth in 2-year-old white spruce seedlings. Asterisks indicate differences (mean) between the breeding strategies using all values from the growth groups (fast, slow) with a Tukey’s test, at 95% confidence level (n=120, 10 blocks, 12 families).

**
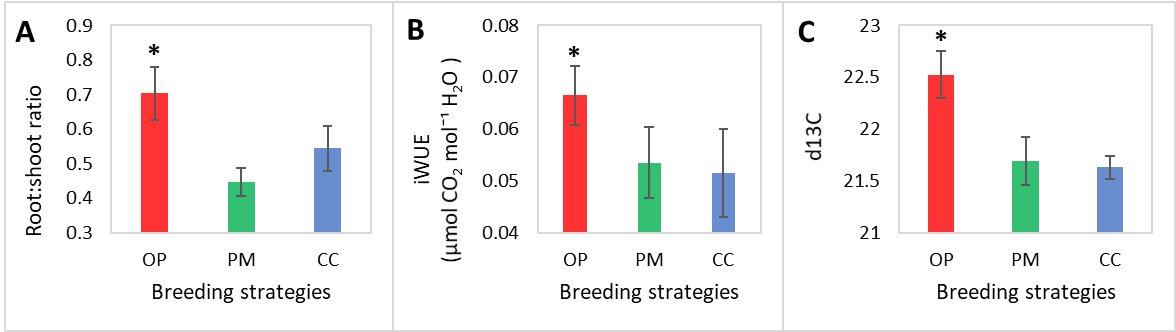
**
